# Focal white matter lesions drive grey matter inflammation and synapse loss

**DOI:** 10.1038/s41586-026-10414-w

**Published:** 2026-04-22

**Authors:** Omar de Faria, Stavros Vagionitis, Andrea Lopez-Lopez, Michael Perry, Joseph Jo Yin Wong, Leslie Rodríguez-Kirby, Bastien Hervé, Balazs Viktor Varga, Eneritz Agirre, Sabrina Ghosh, Sebastian Timmler, Mert Yucel, Andrew T. Setley, Kimberley Anne Evans, Tanja Mist Birgisdóttir, Sindri Gíslason, Yan Ting Ng, Courtney Kremler, Helene O. B. Gautier, Yasmine Kamen, Helena Pivonkova, Katrin Volbracht, Felix Hildebrand, Christian A. Cepeda, Javier Rueda-Carrasco, Soyon Hong, George Malliaras, Sabine Dietmann, Gonçalo Castelo-Branco, Ragnhildur Thóra Káradóttir

**Affiliations:** 1 Cambridge Centre for Myelin Repair, Cambridge Stem Cell Institute & Department of Veterinary Medicine, University of Cambridge, Cambridge, UK; 2 Research Center for Molecular Medicine and Chronic Diseases (CIMUS), University of Santiago de Compostela, CIBERNED, IDIS, Santiago de Compostela, Spain; 3 UCL Great Ormond Street Institute of Child Health, University College London, London, UK; 4 Laboratory of Molecular Neurobiology, Department of Medical Biochemistry and Biophysics, Karolinska Institutet, Stockholm, Sweden; 5 Electrical Engineering Division, University of Cambridge Department of Engineering, Cambridge, UK; 6 Department of Physiology, BioMedical Center, Faculty of Medicine, University of Iceland, Reykjavik, Iceland; 7 UK Dementia Research Institute, University College London, London, UK; 8 Institute for Informatics, Washington University School of Medicine, St Louis, MO USA

**Keywords:** Multiple sclerosis, Alzheimer's disease, Neuroimmunology

## Abstract

Focal white matter lesions occur in most neurodegenerative disorders^1–3^. Despite occurring early in disease, white matter lesions are considered to be independent of, or secondary to, grey matter neuroinflammation, synapse loss and altered neuronal activity^4–7^. Notably, their functional effect on neuronal circuits remains understudied. To address this, we generated a focal white matter lesion in the rat brain within a clinically relevant, anatomically well-defined circuit, in which these lesions occur in many neurodegenerative disorders^8–10^. Here we show that focal white matter lesions evoke transient neuronal activity changes and microgliosis, with subsequent synapse loss and increased microglial engulfment in the grey matter, which is reversed if myelin regeneration completes. Grey matter microgliosis is often considered to be detrimental; however, we show that it is an integral part of regeneration and is conserved across three distinct mouse circuits and lesioning methods. Preventing these transient changes in the grey matter blocks myelin regeneration in the white matter. Conversely, inducing myelin regeneration failure leads to chronic grey matter neuroinflammation. This recapitulates the low-grade inflammation considered to be a dominant mechanism underlying neurodegeneration^7,11,12^. Our findings reveal a form of regenerative plasticity coupling white matter integrity to grey matter function, which may underlie multiple neurodegenerative conditions, and highlight the potential of targeting myelin regeneration to prevent chronic neuroinflammation.

## Abstract (web summary)

A transient grey matter microgliosis is required for remyelination, the failure of which results in a chronic neuroinflammatory state seen in neurodegenerative disorders.

## Main

Focal white matter lesions accumulate with age in the central nervous system (CNS) and, in neurodegenerative conditions, their number correlates with cognitive and physical impairment^1–3,13^. Multiple sclerosis (MS) is a demyelinating disorder characterized by white matter demyelinating lesions^1,7^. Grey matter microgliosis, and synaptic and neuronal functional loss, also occur; however, they are considered independent of lesions and lead to irreversible neurodegeneration, thought to be the dominant mechanism underlying disability progression^5,7^. Neurodegeneration in MS is characterized by low-grade inflammation and ineffective myelin regeneration^7,14^. This is common in other neurodegenerative conditions, such as dementia, Alzheimer’s disease and Parkinson’s disease^1–4^, in which white matter lesions occur early in disease but have been traditionally considered secondary to neuronal loss. However, recent evidence indicates that dysfunctional myelin may affect microglial and neuronal function in mouse models of Alzheimer’s disease^15–17^.

Neurodegenerative disorders, including MS, result in considerable personal, societal and economic burdens, and there are currently no fully effective treatments preventing progression. Changes in neuronal activity^6,18^, microglial activation in the grey matter and synapse engulfment^5,18^ occur in models of diffuse demyelination, and synapse loss and microgliosis are also detected in postmortem tissues from people with MS^5,7,19^ and other neurodegenerative disorders^11^. The observed grey matter changes are often considered independent of the demyelinating injury^5,6,18^, potentially due to the anatomical separation, despite total brain volume loss and cognitive decline correlating with white matter lesion burden^13,20^. The effect of focal white matter lesions on neuronal function and grey matter inflammation remains unclear despite these lesions being a common hallmark of age-related neurodegenerative disorders.

To address this, we induced a focal white matter lesion in an anatomically well-defined circuit and found that it evoked transient and localized grey matter inflammation, synaptic loss and altered neuronal activity, that resolved after white matter regeneration. In contrast to current notions, we found that focal white matter lesions are not secondary to nor independent of, but instead are drivers of, neuronal dysfunction, synapse loss and grey matter inflammation. Rather than being detrimental, these transient effects are an integral part of the regenerative process, with failure leading to ongoing chronic inflammation. This reflects a form of neural plasticity that ensures neuronal health and supports regeneration through coordinated grey–white matter interactions, reframing our understanding of how the nervous system orchestrates regeneration.

## Mapping the circuit after a focal white matter lesion

**Fig. 1:**
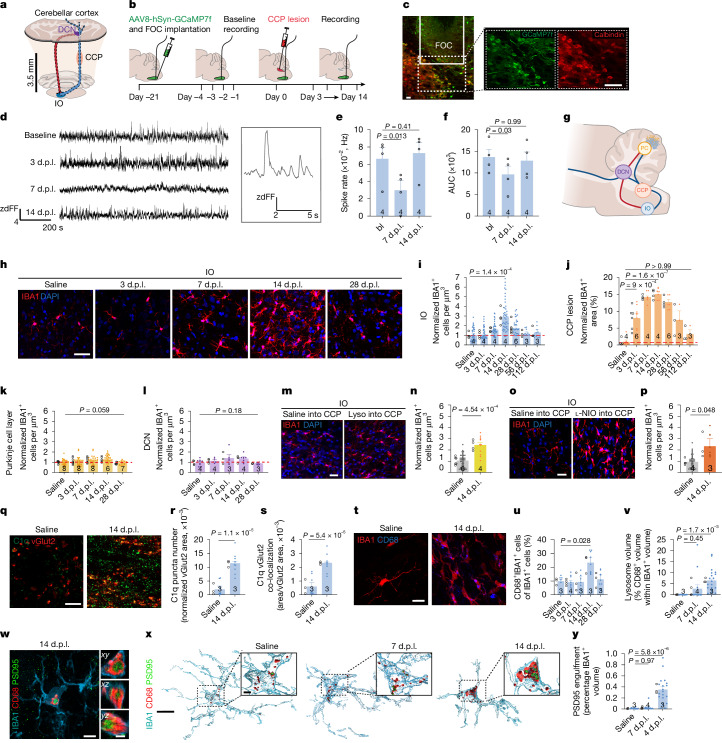
Focal white matter lesions evoke changes in the olivocerebellar circuit. **a**, Focal demyelinating lesions (CCP; red oval) 3.5 mm from the IO (blue circle). Afferent (blue) and efferent (red) fibres run along distinct white matter tracts. **b**, The in vivo fibre photometry recording strategy. **c**, Fibre optic cannula (FOC) placement above GCaMP7f-expressing (green) calbindin^+^ (red) IO neurons confirmed for all rats. *n* = 4 rats. **d**, Fibre photometry recordings (left). Right, enlarged peak. zdFF, *z*-scored delta calcium-dependent fluorescence, calcium-independent fluorescence. **e**,**f**, Reduced spike rate (**e**) and area under the curve (AUC) (**f**) at 7 d.p.l. bl, baseline. **g**, Anatomical regions of the olivocerebellar circuit. Blue, excitatory projections; red, inhibitory projections. **h**–**l**, Imaging (**h**) and quantification (**i**–**l**) showing that IBA1^+^ density increases in the IO (**i**) and lesion (**j**) after focal demyelination but is unchanged in the Purkinje cell layer (**k**) and DCN (**l**). **m**–**p**, CCP lysolecithin (lyso) (**m**,**n**) or L-NIO (**o**,**p**) injection increases the microglial density in the IO (**n**,**p**). **q**–**s**, Immunostaining for C1q (green) and vGLUT2^+^ (red) (**q**) reveals increased C1q^+^ puncta (**r**) and C1q^+^–vGLUT2^+^ co-localization (**s**) at 14 d.p.l. in the IO. **t**–**v**, Immunostaining (**t**) and quantification of IBA1^+^ (red) CD68^+^ (blue) cells (**u**) and microglial lysosome volume (**v**). **w**–**y**, Representative immunostaining (**w**; *n* = 3), 3D reconstruction (**x**) and quantification of engulfed PSD95 (green) within CD68^+^ microglial lysosomes (**y**). Insets: magnified areas. The closed circles represent individual nested measurements: images (**i**–**l**,**n**,**p**,**r**,**s**,**u** and **v**) or cells (**y**) from *n* rats, indicated on the bars. Open circles indicate the rat means. *P* values were calculated using repeated-measures one-way analysis of variance (ANOVA) with Bonferroni post hoc comparisons (**e** and **f**), nested one-way ANOVA with Bonferroni post hoc comparison (**i**–**l**,**u**,**v** and **y**) or nested two-sided *t*-tests (**n**,**p**,**r** and **s**). ANOVA *P* values are provided as Source data. Data are mean ± s.e.m. Scale bars, 2 μm (**w** (right) and **x** (top)), 3 μm (**q**), 5 μm (**w** (left)), 10 μm (**x** (bottom)), 20 μm (**t**), 25 μm (**h**,**m** and **o**), 40 μm (**c** (left)) and 100 μm (**c** (right)). Source data

**Extended Data Fig. 1:**
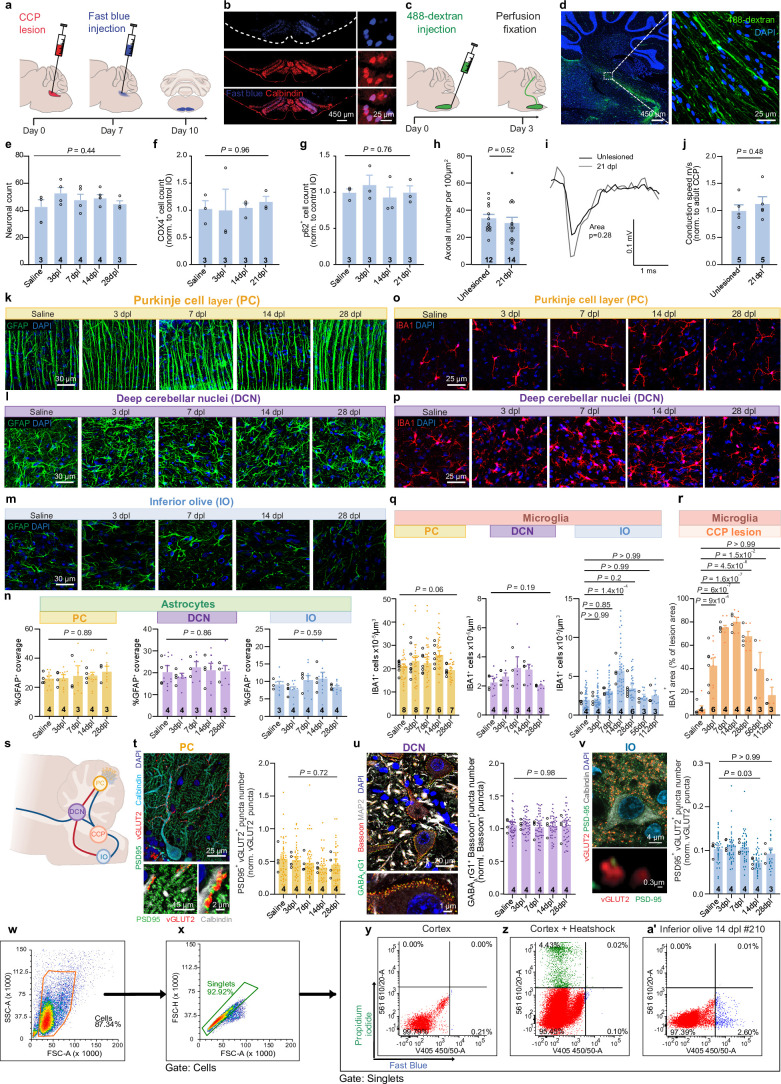
Focal white matter lesions in the CCP evoke changes in the olivo-cerebellar circuit. **a**, Experimental strategy to confirm the identity of neurons projecting through the CCP. Fast blue, a retrograde labelling dye was injected in the CCP at 7dpl followed by perfusion-fixation 3 days later. **b**, Representative (N = 3) image reveals extensive Fast blue (blue) labelling of Calbindin-positive (red) neurons in the IO. **c**, Experimental strategy to identify axonal projections arising from the IO. 488-dextran, an anterograde labelling dye, was injected in the IO and rats perfuse-fixed 3 days later. **d**, Representative (N = 2) image reveals extensive 488-dextran labelling in the CCP. The findings in (**b**) and (**d**) indicate that demyelinated axons in the CCP lesion model are IO projections. **e**, Number of Calbindin-positive neurons in the IO following a focal demyelinating lesion in the CCP. No significant change in the number of CCP-projecting IO neurons was observed across time. **f**,**g**, No significant changes in the number of IO neurons expressing mitochondrial (COX IV) or autophagy (p62) markers were observed across time. **h**, Axonal density is not significantly different between unlesioned and 21dpl CCP. **i**, Traces of compound action potential recordings (N = 5) taken across the unlesioned and 21dpl CCP. The area under the curve is not significantly different between control and 21dpl, indicating that the proportion of axons conducting action potentials is similar between conditions. **j**, Conduction velocity is not significantly different between unlesioned and 21dpl CCP. **k**–**m**, Images containing the PC layer of the cerebellum, DCN and IO stained for the astrocytic marker GFAP. **n**, GFAP-labelled area is not significantly changed in the PC layer, DCN or IO across time. **o**,**p**, Images of PC layer and DCN sections stained for IBA1. **q**, Microglial density is significantly increased at 14dpl in the IO, but not in the PC layer or the DCN. **r**, IBA1-labelled area is transiently increased in the lesion. **s**, Schematic diagram illustrating IO to PC excitatory (blue) inputs, PC to DCN inhibitory (red) inputs and DCN to IO inhibitory inputs (red). **t**, Normalized vGLUT2^+^ PSD95^+^ puncta is not significantly changed in the PC layer following demyelination of the CCP. **u**, Normalized Bassoon^+^ GABA_A_rG1^+^ positive puncta is not significantly changed in the DCN following demyelination of the CCP. **v**, Normalized vGLUT2^+^ PSD95^+^ puncta number is transiently decreased in the IO at 14dpl. **w**, FACS scatter plot. Fast blue was injected in the IO and rats culled 3 days later. Dissociated cells from IO were labelled with propidium iodide for live/dead cell discrimination and Fast blue^+^ cells isolated by FACS. Cells were gated from debris (orange polygon), blue pixels are back gated Fast blue^+^ IO cells. **x**, Singlets were gated from the cells (green quadrangle, red pixels are back gated PI-/FB- cells, green pixels are PI + /FB- cells, blue pixels are PI-/FB+ cells, orange pixels are duplets). Single cells were plotted for Fast Blue (x axis: Ex405, Em 450/50) and propidium iodide (y axis: Ex561, Em610/20). **y**, Cortical cells were used as controls for Fast Blue labelling. **z**, Heat shocked cortical neurons were used as positive controls for propidium iodide labelling. **a’**, Fast blue positive cells (blue pixels) from the IO were negative for propidium iodide and were isolated for transcriptome analysis. Closed circles represent individual nested measurements from images (n, q, r, t-v) from N rats, indicated on the bars. Open circles: animal means. P-values are from one-way ANOVA followed by Bonferroni post-hoc comparisons (e, f, g), nested one-way ANOVA followed by Bonferroni post-hoc comparisons (n, q, r, t, u, v) or two-sided t-test (h, i, j). ANOVA P values are provided in the Source data. Results are given as mean ± s.e.m. Source data

To investigate functional implications of focal white matter lesions for neuronal circuits, we chose the well-defined olivocerebellar circuit for its anatomical separation of afferent and efferent white matter tracts (Fig. 1a). White matter lesions occur in this circuit in MS and other neurodegenerative disorders, such as Alzheimer’s disease, dementia and Parkinson’s disease, and correlate with executive and cognitive deficits^8–10^. We induced a focal white matter lesion in the caudal cerebellar peduncle (CCP) by stereotaxically injecting 0.01% ethidium bromide (EtBr)^21^. The temporal sequence of events within the lesion is well characterized: oligodendrocyte damage and demyelination occur within 3 days post-lesion (d.p.l.), oligodendrocyte precursor cell (OPC) recruitment at 3–7 d.p.l., differentiation into myelinating oligodendrocytes (OLs) at 14 d.p.l. and remyelination by 28 d.p.l.^21^. Axonal integrity is preserved: demyelinated axons conduct action potentials^21^ with no significant reduction in the proportion of conducting axons, and ultrastructural analysis reveals unchanged axonal density compared to unlesioned CCP (Extended Data Fig. 1h–j). To trace neuronal cell bodies of demyelinated axons, we injected retrograde dye (for example, Fast Blue) into the CCP, revealing that labelled axons originate from calbindin^+^ projection neurons in the inferior olive (IO; 64.1 ± 5.9% IO neurons), a nucleus in the medulla 3.5 mm from the lesion (Fig. 1a and Extended Data Fig. 1a,b). Anterograde dye (for example, dextran) injection into the IO confirmed calbindin^+^ projection neurons project through the CCP (Extended Data Fig. 1c,d). After lesioning, no loss or damage of IO calbindin^+^ neurons was detected (Extended Data Fig. 1e–g,w–a′). This model provides a clear in vivo reductionist approach to dissect the consequences of white matter lesions on circuit function.

## Neuronal activity transiently decreases after lesion

Neuronal activity is important for myelin regeneration^21,22^, but a direct link to focal white matter lesions has not been investigated. To test this, we transduced demyelinated IO neurons (Fig. 1b) with the calcium indicator GCaMP7f (AAV8-hSyn-GCaMP7f; 96.3 ± 1.4% GCaMP7f^+^ IO cells are calbindin^+^). We used in vivo photometry to record calcium changes in the IO as a proxy for action potential firing^23^, in freely moving rats before and every 2–4 days after CCP lesioning, throughout the regenerative process (Fig. 1c,d). Our data show a transient 60% decrease in firing rate after lesion, reaching the lowest levels at around 7 d.p.l. (Fig. 1e,f). This reduction is unlikely to be due to dysregulated metabolism or cell death, as ATP depletion initially increases neuronal activity^24^. Nor can long-term suppression be explained by homeostatic responses of this negative feedback circuit, as reduced climbing fibre (CF) input to Purkinje cells causes IO disinhibition^25^. By the onset of remyelination, IO neuronal activity returns to pre-lesion levels (Fig. 1e,f), indicating a transient adaptive neuronal response to focal demyelination.

## Cellular changes in the circuit following lesion

**Extended Data Fig. 2:**
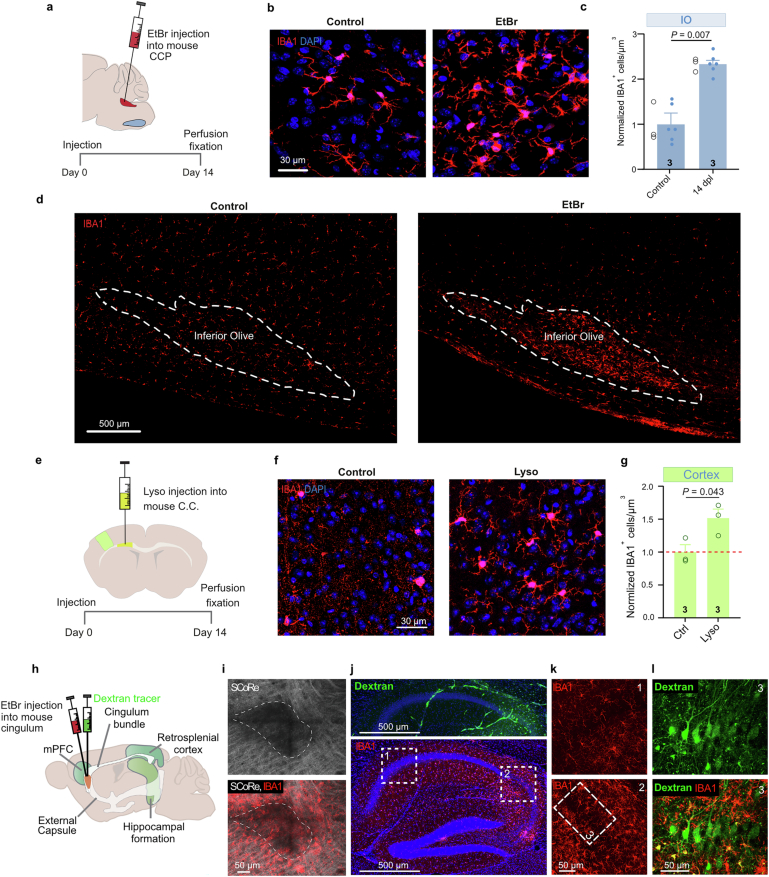
White matter lesions evoke a microglial response in the grey matter irrespective of model, circuit and species. **a**, Schematic showing the experimental design for EtBr lesioning of the mouse CCP. **b**,**c**, Mouse IO sections stained for IBA1 (red) and DAPI (blue) (b). Like the observations in the rat CCP model, microglial density is elevated in the IO at 14dpl (c). **d**, Representative (N = 3) low magnification images of mouse brainstem stained for IBA1 showing that the increase in microglia density in the IO is pronounced and local. **e**, Schematic showing the experimental design for lysolecithin lesioning of the mouse corpus callosum. **f**, Mouse cortical sections stained for IBA1 (red) and DAPI (blue). **g**, Microglial density is increased at 14dpl in the cortex, indicating that a similar microglial response develops in the lysolecithin demyelinating model. **h**, Schematic showing the experimental design for EtBr lesioning in the mouse cingulum bundle. **i**, Representative mouse sections imaged using spectral confocal reflectance microscopy (SCoRe; N = 3) and stained for IBA1 (red). Lesion outlined with dotted line. **j**, Representative (N = 2) mouse hippocampal sections stained for IBA1 (red), DAPI (blue) and labelled with Dextran (green). **k**, (top) Magnification of box 1 shown in **j**. (Bottom) Magnification of box 2 shown in **j**. **l**, Magnification of box 3 shown in **k** highlights that microglia density is increased around Dextran^+^ neurons, suggesting that a similar microglial response develops in the hippocampus following demyelination of the cingulum. Closed circles in bar graphs represent individual nested measurements from images from N number of mice, indicated on the bars. Open circles: animal means. P values are from nested two-sided t-test (c) and two-sided t-test (g). Results are given as mean ± s.e.m. Source data

After peripheral nerve injury, we and others have reported remote retrograde activation of CNS glia around neuronal somata of injured peripheral axons^26,27^. Grey matter gliosis has also been described in diffuse demyelination and experimental acute encephalomyelitis^5–7,18^. However, it remains unclear whether a focal white matter demyelinating injury that leaves neurons functionally intact can elicit grey matter glial cell changes. To address this, we used the anatomically defined olivocerebellar circuit to map glial changes downstream (cerebellar cortex Purkinje cell layer, deep cerebellar nuclei (DCN)), and upstream (IO), before and after lesioning the CCP (Fig. 1g). We detected no significant changes in glial fibrillary acidic protein (GFAP), expressed in CNS astrocytes and Bergmann glia, and elevated in astrogliosis^28^, around CF–Purkinje-cell terminals, the DCN or the IO (Extended Data Fig. 1k–n). By contrast, microglial density (quantified as cells positive for IBA1)^29^ increased at 7 d.p.l., peaking at 14 d.p.l. with a threefold increase in the IO compared with in unlesioned rats (Fig. 1h,i). No significant changes were detected in adjacent medullary regions, CF–Purkinje cell terminals or the DCN (Fig. 1k,l and Extended Data Fig. 1o–q), demonstrating localized responses around IO neurons (Extended Data Figs. 2d and 8b). This grey matter microgliosis, evoked by focal white matter demyelination, followed the inflammation within the lesion and resolved after remyelination (Fig. 1i,j and Extended Data Fig. 1q,r).

## Grey matter microgliosis across lesion models

The observed grey matter inflammation is not specific to demyelinating agent, circuit or species. Focal CCP demyelination with EtBr, lysolecithin or by focal blood vessel occlusion, using endothelial nitric oxide synthase inhibitor *N*^5^-(1-iminoethyl)-L-ornithine (L-NIO)^30^, mimicking hypoxic white matter focal injury in age and neurodegenerative disease, all equally increase IO microglial density at 14 d.p.l. (Fig. 1m–p). Nor is the microglial response unique to the olivocerebellar circuit, or rats, as similar changes are detected in the mouse cortex after a lysolecithin-induced corpus callosum lesion, in the hippocampus after cingulum lesioning and in the IO after EtBr injection into the mouse CCP (Extended Data Fig. 2). After remyelination, microglial density returns to control levels throughout the circuit, although a non-significant elevation persists in the CCP (Fig. 1j). This transient grey matter microglial response around demyelinated neurons represents a generic microglial response to focal white matter lesions that resolves after remyelination.

## Synapse loss at dendrites of demyelinated neurons

Synapse loss alongside grey matter microgliosis has been reported in MS post-mortem tissue and animal models, reminiscent of changes detected in dementia^7^. To determine whether focal white matter lesions evoke grey matter synapse loss, we quantified post-lesion synapse numbers across the circuit. No changes were detected at CF–Purkinje-cell terminals (excitatory) (Extended Data Fig. 1s,t) or at Purkinje–DCN synapses (GABAergic) (Extended Data Fig. 1s,u). By contrast, excitatory synapses on calbindin^+^ IO neurons transiently decreased (Extended Data Fig. 1s,v). This coincided with increased complement component 1q (C1q) deposition at synaptic terminals (Fig. 1q–s), a key component of the complement pathway reported to regulate microglial synaptic engulfment^31^, and with an elevated fraction of CD68^+^ microglia displaying enlarged lysosomes (Fig. 1t–v) containing excitatory synaptic material (Fig. 1w–y and Supplementary Videos 1–3). IO excitatory synapses were transiently and partially lost at 14 d.p.l., before returning to control levels at 28 d.p.l. (Extended Data Fig. 1v), when remyelination completes.

## Distinct transcriptional changes within the circuit

**Fig. 2:**
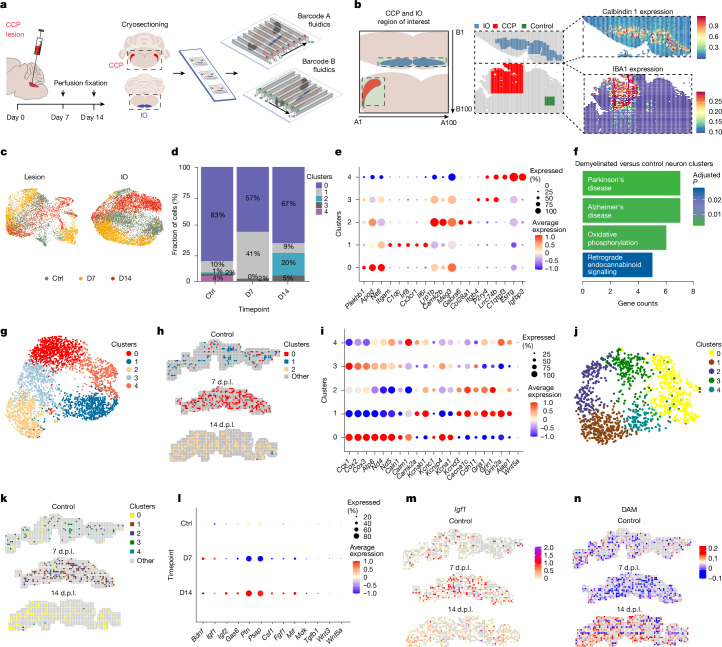
Spatial transcriptomics reveal distinct responses in the IO and lesion over time. **a**, The experimental strategy for DBiT-seq. **b**, Representative examples (*n* = 3 for lesions and *n* = 4 for the IO) of DBiT-seq spatial readout. Calbindin 1 expression was used to delineate the IO; IBA1 expression was used to identify the lesion. **c**, UMAP of pixels from lesion (left) and IO (right), coloured by timepoint. **d**, Lesion microglial cluster 1 is enriched at 7 d.p.l. and cluster 2 at 14 d.p.l. **e**, Differential gene expression (DGE) analysis of different microglial signatures in lesion microglial clusters. **f**, Gene Ontology (GO)-term analysis of genes differentially expressed between demyelinated and control IO neuron clusters reveals terms associated with Alzheimer’s disease, Parkinson’s disease and mitochondrial metabolism. **g**, Five neuronal clusters were identified by unsupervised clustering of calbindin 1^+^ pixels in the IO. **h**, IO neuronal cluster 0 is enriched at 7 d.p.l. and cluster 2 at 14 d.p.l. **i**, DGE analysis of different neuronal clusters in the IO. **j**, Five microglial clusters were identified by unsupervised clustering of microglial pixels in the IO. **k**, IO microglial clusters 1 and 2 are enriched at 7 d.p.l. and microglial cluster 0 at 14 d.p.l. **l**, DGE analysis of neuroprotective markers in the IO microglia over time. **m**, IGF1 expression is increased in IO microglia at 7 d.p.l. **n**, Expression of disease-associated microglia (DAM, reported previously^33^) genes is increased in the IO at 14 d.p.l. Data are from *n* = 3 (lesion analysis) and *n* = 4 (IO analysis) rats. GO-term analysis *P* values were derived from one-tailed Fisher’s exact tests with Benjamini–Hochberg false-discovery rate multiple-testing correction. Exact* P* values for the GO analysis are provided in Supplementary Table 1. Source data

To gain further insights into how focal white matter lesions cause these changes, we used spatial transcriptomics using deterministic barcoding in tissue (DBiT-seq)^32^. This enables simultaneous analysis of cellular transcripts in demyelinating lesions in the CCP and the associated IO (Fig. 2a).

### Microglia in focal demyelinated lesions

**Extended Data Fig. 3:**
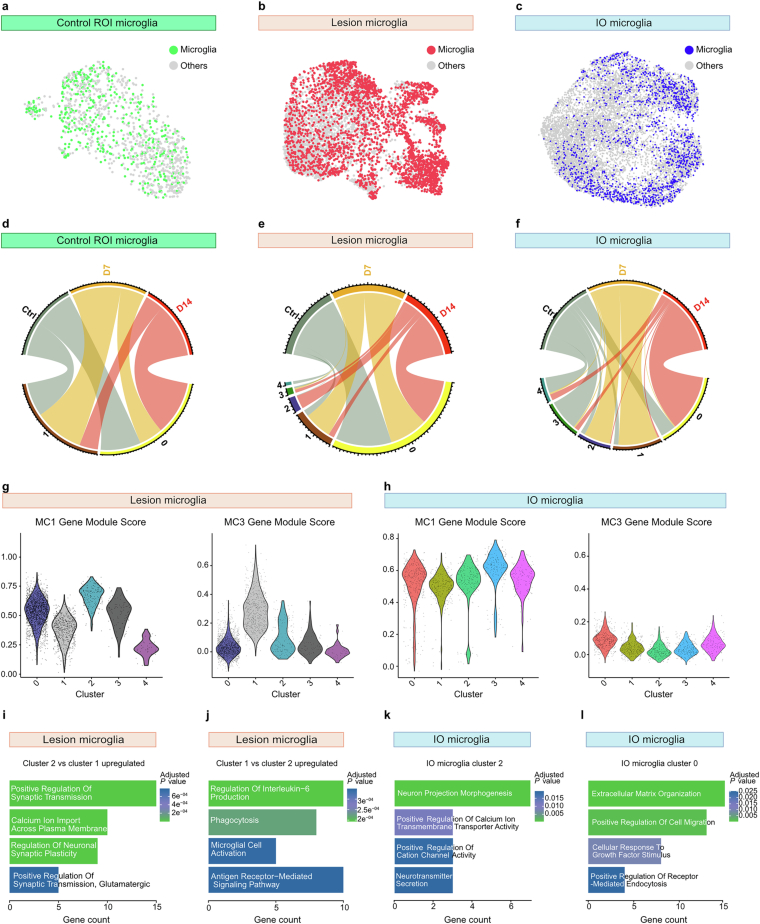
Spatial transcriptomic analysis identifies distinct subsets of microglia in healthy control area, lesion and IO following demyelination in the CCP. **a**, UMAP of all microglia pixels called in the control ROI. **b**, UMAP of all microglia pixels called in the CCP lesion. **c**, UMAP of neuron-associated microglia pixels called in the IO. **d**, Chord plot showing the relative contribution of each control ROI microglia cluster to the different timepoints. **e**, Chord plot showing the relative contribution of each lesion microglia cluster to the different timepoints. **f**, Chord plot showing the relative contribution of each IO microglia cluster to the different timepoints. Data in **d**, **e** and **f** indicate that changes in microglia subcluster composition develop exclusively in the CCP and IO, with no significant changes observed in control health areas. **g**, Violin plots showing MC1 (left) and MC3 (right) gene module score in lesion microglia. **h**, Violin plots showing MC1 (left) and MC3 (right) gene module score in IO microglia. Data in **g** indicates that lesion microglia clusters 2 and 1 share similarities with microglial clusters MC1 and MC3, identified in lysolecithin demyelinating lesions by Zhang, Rodriguez-Kirby et al., 2025. None of the IO microglia clusters, however, are particularly similar to MC1 or MC3. **i**, GO-term analysis of markers of lesion microglia cluster 2. **j**, GO-term analysis of markers of lesion microglia cluster 1. Analysis in **i** and **j** suggest an inflammatory/phagocytic phenotype for lesion microglia cluster 1 and a neuron-supporting profile for lesion microglia cluster 2. **k**, GO-term analysis of IO microglia cluster 2 markers. **l**, GO-term analysis of IO microglia cluster 0 markers. Analysis in **k** and **l** indicate a neuroprotective phenotype for cluster 2 and a mixed profile, comprising both neuroprotective and inflammatory signatures, for cluster 0. Data is from N = 3 rats for the lesion analysis and N = 4 rats for the IO analysis. GO term analysis P values derive from one-tailed Fisher’s exact test with multiple testing corrected by Benjamini–Hochberg false discovery rate. Exact P values for GO analysis are shown in Supplementary Table 1. Source data

We identified pixels belonging to CCP lesions by IBA1 immunostaining of corresponding sections (total 6,271 pixels, from 9 CCPs; Fig. 2b,c). Within these lesions, we analysed pixels containing microglial signatures (positive for *Aif1*,* Maf*,* Csf1r*,* Spi1*; 2,874 pixels identified), revealing five microglial clusters (Fig. 2d and Extended Data Fig. 3a,b,e), some resembling previously identified clusters in focal demyelinating lesions^32^ (Extended Data Fig. 3g). Cluster 1, a pro-inflammatory and phagocytotic-like cluster, predominates at 7 d.p.l. (Fig. 2d,e and Extended Data Fig. 3e,g,j), when myelin removal nears completion and OPC proliferation peaks. At 14 d.p.l., when OPC differentiation begins, cluster 2 becomes prominent, resembling a previously defined neuron and myelin supportive microglial cluster^32^ (Fig. 2d,e and Extended Data Fig. 3g,i). The similarities to previous data, despite differences in lesion induction, brain regions, white matter tract and species, further demonstrate that, independent of how focal white matter lesions occur, there are common cellular responses that initiate myelin clearing, OPC proliferation and differentiation that are necessary for myelin regeneration.

### Focal white matter lesions evoke transcriptional changes in the IO

We further investigated the changes in the IO, focusing on neurons and microglia, as these showed significant changes in our physiological and histological data (Fig. 1), and followed demyelination and regeneration in the lesion.

**Extended Data Fig. 4:**
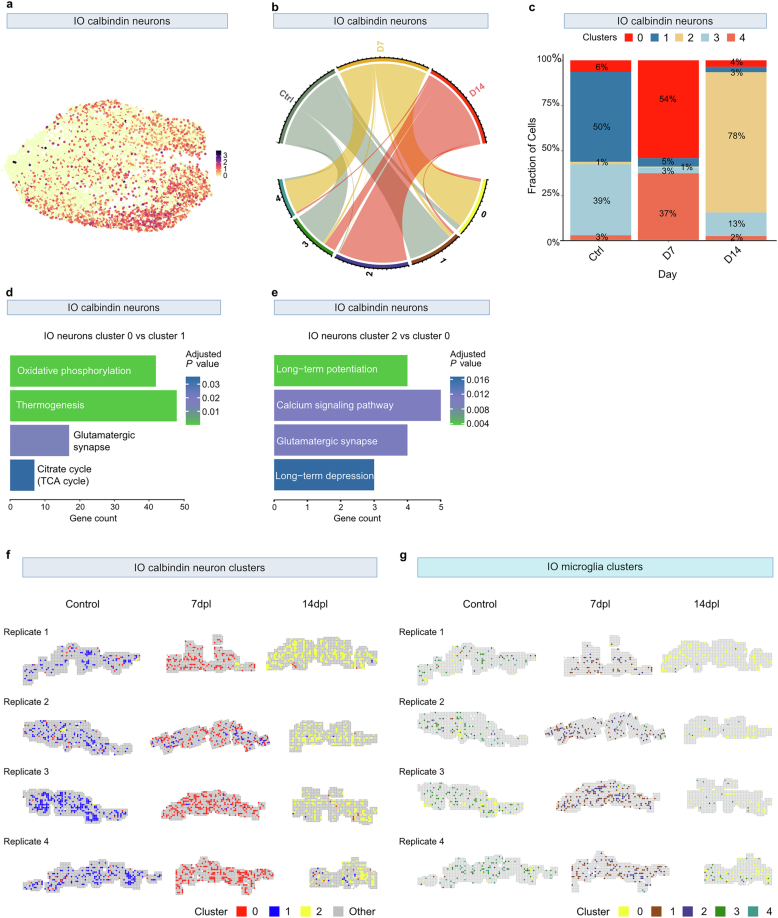
Spatial transcriptomic analysis identifies distinct neuronal clusters in the IO following demyelination of the CCP. **a**, UMAP plot highlighting all calbindin-positive pixels in the IO. **b**, Chord plot showing the relative contribution of each IO calbindin neuron cluster to the different timepoints. **c**, IO neuron cluster distribution across time points. Five clusters were identified in the IO. Clusters 1 and 3 are prominent in controls, whereas clusters 0 and 4 emerge at 7dpl and cluster 2 at 14dpl. **d**, GO-term analysis of differentially expressed genes between markers of clusters 0 and 1 highlights changes in mitochondrial metabolism and neuronal excitability. **e**, GO-term analysis of differentially expressed genes between markers of clusters 2 and 0 shows gene signatures related to membrane potential regulation, excitability and synaptogenesis. **f**,**g**, Every replicate of the cluster tissue maps shown in Fig. 2. Neuronal cluster 1 predominates in the control IO, while clusters 0 and 2 are the most abundant at 7 and 14dpl, respectively. Microglia clusters 1 and 2 are consistently increased at 7dpl in the IO, whereas cluster 0 is the most prevalent at 14dpl. Data is from N = 4 rats GO term analysis p values derive from one-tailed Fisher’s exact test with multiple testing corrected by Benjamini–Hochberg false discovery rate. Exact P values for GO analysis are shown in Supplementary Table 1. Source data

IO neurons were identified by calbindin^+^ pixels (3,442 out of 10,864 pixels representing the IO region; Fig. 2b,c and Extended Data Fig. 4a). Demyelinated IO neurons show no detrimental cell stress associated with cell death or neurodegeneration, nor increased propidium iodide positivity or other markers of damage (Extended Data Fig. 1w–a′). Instead, we detected gene expression signatures indicating an adaptive response to the demyelinating lesion, including genes associated with axonal transport (*Kif13a*,* Kif13b*,* Kif5c*), membrane potential regulation (*Kcnab1*,* Kcnc1*,* Kcnd3*) and metabolic function (*Cox1*,* Atp6*). KEGG pathway analysis revealed enrichment of gene set signatures related to oxidative phosphorylation, retrograde endocannabinoid signalling, Alzheimer’s disease and Parkinson’s disease (Fig. 2f).

Subclustering of calbindin^+^ IO pixels revealed five subclusters (IO0–4; Fig. 2g,i). Cluster IO0, prominent at 7 d.p.l. (Fig. 2h and Extended Data Fig. 4b–d), displayed changes in mitochondria biogenesis (*Cox1*, *Cox2*, *Cox3*,* Nd2*,* Atp6*), calcium buffering (*Calm1*,* Calb1*), neuronal hyperpolarization (*Kcnj16*,* Kcnj3*) and excitability (*Kcnc3*,* Kcnc2*,* Scn4b*) (Fig. 2i and Extended Data Fig. 4d). Cluster IO4 exhibited IO0-like signatures and overlapped with control clusters IO3 and IO1, suggesting a transitional state between control and demyelinating neurons (Fig. 2i). Cluster IO2 was enriched at 14 d.p.l., showing gene signatures for membrane potential regulation and excitability (*Cacna1*,* Kcnab1*,* Kcnd3*), synaptogenesis (*Cdh11*,* Ajap1*,* Slit3*,* Synpo2*) and neurotransmission (*Gria1*,* Grin1*) (Fig. 2h,i and Extended Data Fig. 4c,e). Collectively, these subclusters and pathway analyses suggest that, after demyelination, neurons initially maintain energy by reducing excitability and augmenting mitochondria biogenesis and calcium buffering. This is followed by activation of pathways supporting maintenance and restoration of neuronal function, consistent with a coordinated adaptive response that maintains cellular function and integrity during myelin regeneration (Extended Data Fig. 4d,e).

### Microglial transcriptomic signatures change around demyelinated neurons

**Extended Data Fig. 5:**
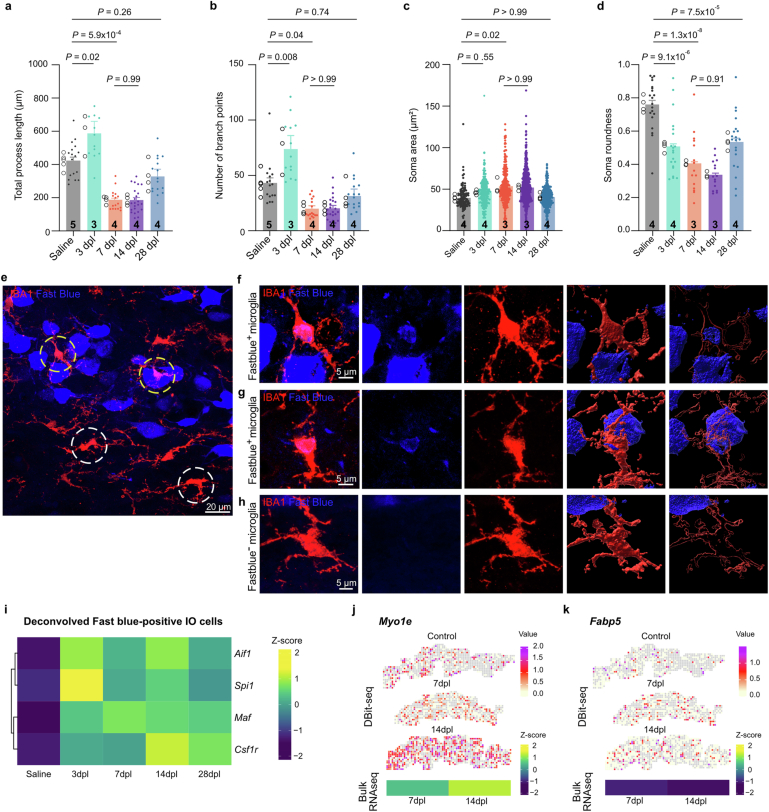
Cell morphology and transcriptomic analysis identify distinct microglial cell states. **a**,**b**, Total process length (a) and mean number of branch points (b) is significantly increased at 3dpl and decreased at 7 and 14dpl. **c**, Microglial cell body size is increased at 7 and 14dpl. **d**, Microglial cell body roundness is decreased at 7 and 14dpl. **e**, Fast blue, a retrograde labelling dye, was injected in the lesioned CCP and the IO processed for IBA1 staining. Representative (N = 2) low-magnification image shows fast blue-labelled demyelinated neurons (large blue cells) and fast blue-positive (yellow circles) and negative (white circles) microglia. **f**,**g**, Examples (N = 2) of a subset of IBA1-positive (red) inferior olive microglia which interact closely with fast blue-positive (i.e., demyelinated) neurons and display internalization of the same dye (blue). Fast blue-labelled microglia are not detected in saline controls. **h**, Representative (N = 2) IBA1-positive (red) microglial cell that surveys the environment but does not interact closely with fast blue-positive neurons and is not labelled with fast blue (blue). **i**, Heatmap showing expression of four microglia markers in the bulk RNAseq deconvolved for microglia signatures. These markers were used to identify microglia-positive pixels in the spatial transcriptomic analysis. Data are z-score normalized. **j**,**k**, DBit-Seq and bulk RNAseq show the same pattern of gene expression. Myo1e and Fabp5 are shown for comparison. Dbit-seq tissue maps are representative of N = 4 rats. Bulk RNAseq differential expression analysis is from N = 3 rats. In bar graphs, closed circles represent individual nested measurements from cells from N rats, indicated on the bars. Open circles**:** animal means. P values are from nested one-way ANOVA followed by Bonferroni t-test. Results are presented as mean ± s.e.m. Source data

Our data indicate that IO microglial density increases after a CCP demyelinating lesion, peaking at 14 d.p.l. (Fig. 1i) when they phagocytose synapses (Fig. 1). Moreover, microglia near demyelinated neurons become Fast Blue labelled from 3 d.p.l., in contrast to the unlesioned controls, suggesting tight interactions between a subset of microglia and cell bodies of demyelinated neurons (Extended Data Fig. 5e–h). Subclustering 1,201 pixels containing microglial signatures (*Aif1*,* Maf*,* Csf1r*,* Spi1*) near calbindin^+^ pixels identified five subclusters: mIO0–4 (Fig. 2j,k and Extended Data Fig. 3c,f). Subcluster 2, prominent at 7 d.p.l., expresses neurotrophic factors (*Bdnf*,* Igf1*, *Fbxw7*) (Fig. 2k–m and Extended Data Fig. 3c,f,h,k). At 14 d.p.l., cluster 0 is prominent, with a complex neuroprotective phenotype (*Igf2*, *Gas6*,* Ptn*,* Psap*,* Ptgds*), and microglial signatures associated with neurodegenerative disease^33^ (*Apoe*,* Spp1*) (Fig. 2k,n and Extended Data Fig. 3l). These IO microglial clusters differ markedly from those in the lesion and in healthy control areas, demonstrating location-dependent responses with distinct functional phenotypes after the same insult. Although increased microglial density with synapse loss is conventionally attributed to neuronal damage, our spatial transcriptomic analysis suggests microglia are neuroprotective and actively contribute to regeneration.

## Distinct microglial states in the grey matter

**Fig. 3:**
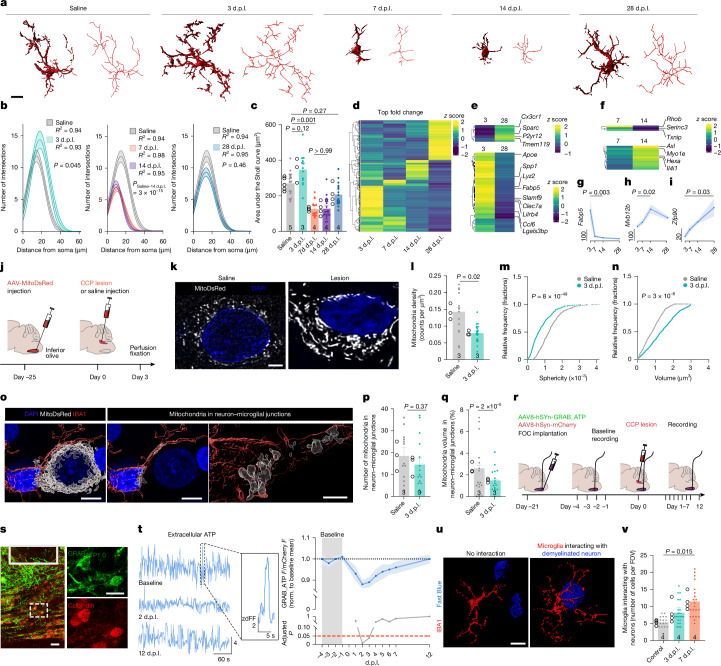
Focal white matter lesions evoke changes in microglial states and neuronal mitochondria. **a**, Three-dimensional microglia reconstructions shown as surfaces (left) and filaments (right). **b**, Gaussian fits of the Sholl analysis. The middle solid line indicates the mean, and the shaded area represents the 95% confidence intervals. **c**, The area under the Sholl curve. **d**–**f**, DGE analysis showing the top 200 differentially expressed gene compared to 28 d.p.l (**d**), and selected differentially expressed genes between 3 and 28 d.p.l. (**e**) and between 7 and 14 d.p.l. (**f**). **g**–**i**, Gene expression patterns of *Fabp5* (**g**), *Mvb12b* (**h**) and *Zfp90* (**i**). **j**, The experimental strategy for genetically labelling neuronal mitochondria in the IO. **k**, Images showing MitoDsRed-labelled mitochondria. **l**–**n**, Neuronal mitochondrial density (**l**) and sphericity (**m**) are decreased whereas the volume (**n**) is increased at 3 d.p.l. **o**, Three-dimensional reconstructions of total neuronal mitochondria (left) and mitochondria within microglial–neuron junctions (middle and right). **p**,**q**, The mitochondrion number (**p**) in microglia–neuron junctions is unchanged, but the mitochondrial volume (**q**) is reduced at 3 d.p.l. **r**, The experimental strategy for recording neuron-derived extracellular ATP using fibre photometry. **s**, Immunostaining (*n* = 5) confirming GRAB_ATP1.0_ (green) expression in calbindin^+^ (red) neurons. **t**, Fibre photometry recordings (*n* = 5; left) and summary data (right). Data are mean ± s.e.m. *P* values are plotted per timepoint of recording at the bottom right. **u**, Three-dimensional reconstructions of microglia and Fast-Blue-labelled neurons. **v**, The number of microglia closely interacting with neurons. FOV, field of view. The closed circles represent individual nested measurements: cells (**c**), mitochondria (**l**,**m** and** n**), neuron–microglial junctions (**p**,**q**) or images (**v**) from *n* rats, indicated on the bars. The open circles indicate rat means. *P* values were calculated using two-sided Wald *z*-tests with Holm correction (**b**), nested one-way ANOVA followed by Bonferroni post hoc comparisons (**c** and **v**), Wald tests (two-sided) followed by Benjamini–Hochberg correction (**d**–**i**), nested two-sided *t*-tests (**l**), two-sided Kolmogorov–Smirnov tests (**m** and **n**), two-sided hierarchical bootstrap analysis (**p** and **q**) and mixed-effect model followed by Bonferroni post hoc comparisons (**t**). ANOVA *P* values are provided as Source data. Data are mean ± s.e.m. Norm., normalized. Scale bars, 2 μm (**k** and **o** (right)), 5 μm (**o** (left and middle) and **u**), 10 μm (**a**) and 30 μm (**s**). Source data

Microglia respond to injury or disease by changing state, characterized by changes in transcriptomic signature and morphology. Three-dimensional reconstructions and Sholl analysis of microglia surrounding demyelinated IO neurons after a focal CCP lesion showed progressive morphological changes. At 3 d.p.l., microglia became hyper-ramified with wider coverage, more processes and branches (Fig. 3a–c and Extended Data Fig. 5a,b), a morphology that is often associated with increased surveying^29,34^. By 7 d.p.l., they began decreasing in size, covering reduced area and having shorter processes, fewer branches and larger less rounded soma (Fig. 3a–c and Extended Data Fig. 5a–d). Microglia reach their smallest size at 14 d.p.l., resembling phagocytic microglia^29,34^. Once remyelination in the CCP was complete at 28 d.p.l., IO microglia returned to control-like morphology.

**Extended Data Fig. 6:**
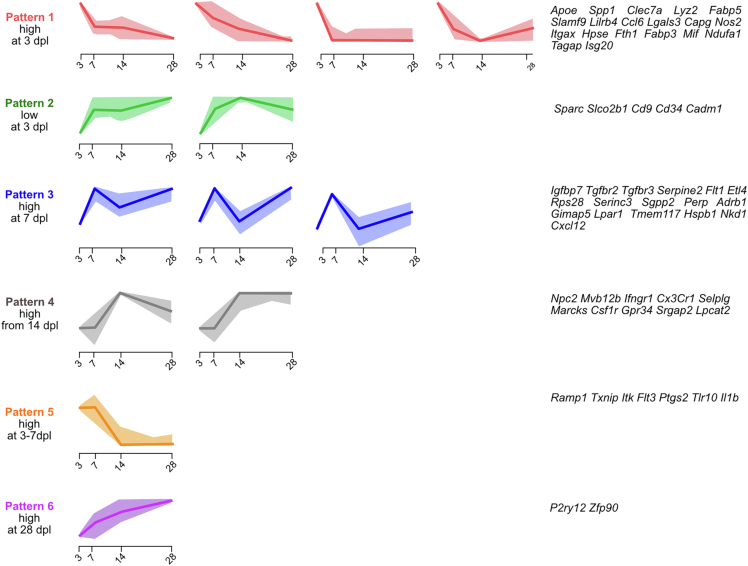
Mapping of deconvolved IO microglia signatures over time identifies 6 patterns of gene expression. Several genes associated with activated microglia (selected genes listed on the right) follow pattern 1 of gene expression, being highly expressed at 3dpl and expressed at lower levels thereafter. Several genes associated with homeostatic microglia follow pattern 2, being expressed at low levels at 3dpl, or pattern 4, showing low expression up to 7dpl and higher levels at 14 and 28dpl. Neuroprotective genes, such as Igfbp7, Tgfbr2 and Tgfbr3 follow pattern 3, peaking expression at 7dpl. Genes following pattern 5 (e.g. Ramp1) are highly expressed at 3 and 7dpl but subside thereafter. Other homeostatic genes, such as P2y12r and Zfp90 show low expression at 3dpl and high at 28dpl (pattern 6). Data is from 3 rats for each time point. Bold lines represent the mean ± s.e.m. (shaded area). Source data

To examine whether microglial morphological states have distinct transcriptional signatures, and improve the temporal resolution, we deconvolved microglia-specific signatures from bulk RNA-sequencing (RNA-seq) analysis of Fast-Blue-positive IO cells (Extended Data Fig. 5i) and analysed the subset of microglia directly interacting with demyelinated neurons. We observed temporal gene expression changes (Fig. 3d and Extended Data Fig. 6), with microglial activation signatures at 3 d.p.l. (for example, *Spp1*, *Clec7a*, *Fabp5*, *Lgals3*; Fig. 3e,g and Extended Data Fig. 6 (patterns 1 and 2)). Most activation-related expression subsided by 7 d.p.l., when neuroprotective signatures were elevated in the bulk dataset (for example, *Igfbp7*; Extended Data Fig. 6 (pattern 3)) and prominent in the spatial data (Fig. 2k–m and Extended Data Fig. 3k). At 14 d.p.l. a subset of disease-associated microglia markers (such as *Myo1e*,* Axl*, *Hexa*, *Il4i1*; Fig. 3f), additional inflammatory or phagocytic markers (for example, *Innir1*,* Npc2*,* C3*,* Mvb12b*; Fig. 3h and Extended Data Fig. 6 (pattern 4)) and homeostatic genes (such as *Cx3Cr1*,* Selplg*,* Marcks*, *Csf1r*,* Gpr34*, *Srgap2*; Extended Data Fig. 6 (pattern 4)) were expressed, consistent with the emergence of the mixed-signature mIO cluster 0 in the spatial transcriptomics analysis (Fig. 2k–n). After remyelination, genes associated with suppression of microglia activation (for example, *Zfp90*; Fig. 3i) and homeostasis (such as *P2y12r*) were upregulated (Extended Data Fig. 6 (pattern 6)). Temporal signature changes were consistent between deconvolved and spatial datasets at shared timepoints (Extended Data Fig. 5j,k). Collectively, these morphological and transcriptomic changes suggest that focal white matter lesions evoke transient microglial states as early as 3 d.p.l., which then return towards a homeostatic state once myelin regeneration is underway in the CCP.

## Mitochondrial networks remodel in neurons

The microglial response is intriguing given that demyelination is focally induced millimetres away from the IO (Fig. 1a), too far for chemical diffusion^35^ and too spatially restricted for blood borne factors (Extended Data Figs. 2d and 8b), yet proximate to demyelinated neurons. Transcriptomic analysis revealed a distinct subcluster of demyelinated IO neurons with altered mitochondrial and calcium-buffering genes (Fig. 2g–i and Extended Data Fig. 4c,d), indicating mitochondrial remodelling after a lesion. Combined with previous findings of increased mitochondrial trafficking from neuronal somata to axons after demyelination^36,37^, we reasoned that altered mitochondrial dynamics in demyelinating neurons might represent an initial grey matter response to demyelination that activates IO microglia.

To test this, we expressed a mitochondria-specific fluorescent reporter (pAAV2-CAG-MitoDsRed-IRES-GFP) in IO neurons 3 weeks before CCP lesion induction (Fig. 3j). At 3 d.p.l., demyelinated neuronal somata contained fewer mitochondria, potentially indicating increased mitochondria trafficking down the axon (Fig. 3k,l).

Mitochondria homeostatically remodel their shape through fusion or fission according to neuronal energy demand and injury response. Increased fission often signals cellular stress, as in neurodegenerative conditions such as Alzheimer’s disease^38^ or stroke^39^, in which mitochondria appear small and rounded. By contrast, mitochondria in demyelinated neurons at 3 d.p.l. were elongated relative to controls, suggesting increased fusion, resulting in fewer but larger mitochondria (Fig. 3m,n). This indicates healthy mitochondrial remodelling toward effective oxidative phosphorylation and internal ATP production, consistent with the neuronal IO0 cluster profile (Fig. 2h,i and Extended Data Fig. 4c,d).

## Lesions alter neuron–microglial ATP signalling

Homeostatic microglia continuously survey neurons by sensing ATP release, for example, at somatic purinergic junctions, where microglial processes closely appose neuronal mitochondria^39^. We detect similar junctions on demyelinated and unlesioned IO neurons, but the mitochondrial content at these junctions is significantly reduced at 3 d.p.l. compared with in the unlesioned controls (Fig. 3p,q). To assess ATP signalling between demyelinated neurons and microglia, we expressed the extracellular ATP biosensor GRAB_ATP_1.0, a modified human P2Y receptor with permutated enhanced GFP, in IO neurons enabling real-time sensing of ATP release (Fig. 3r,s). Before the lesion, we detected homeostatic neuronal ATP release, which began declining at 1 d.p.l., with a significant 13.2 ± 1.3% reduction by 2 d.p.l., preceding reduced neuronal activity (Figs. 1e,f and 3t). Extracellular ATP remained significantly reduced for 2 days before gradually returning to control levels by 12 d.p.l., coinciding with the recovery of neuronal activity. These temporal changes in extracellular ATP aligned with a shift towards surveillance-like microglial morphology (Fig. 3a–c and Extended Data Fig. 5a–d) and activated gene expression at 3 d.p.l. (Fig. 3d,e,g and Extended Data Fig. 6), consistent with ATP-dependent regulation of microglia at these junctions^39,40^.

Disruption of somatic neuron–microglial junctions and altered extracellular ATP signalling promote neuroprotective microglial–neuron interactions^39,41^. Quantification of microglial contacts (defined as microglial processes or cell bodies enwrapping or touching neuronal somata) with demyelinated IO neurons revealed increased interactions with calbindin^+^ neurons at 7 d.p.l. (Fig. 3u,v), when neuronal activity reached the minimum (Fig. 1e,f). This aligns with increased Fast Blue^+^ microglia in close proximity to demyelinated neurons (Extended Data Fig. 5e–h) and the emergence of the neuroprotective mIO2 cluster at 7 d.p.l. (Fig. 2k–n and Extended Data Fig. 3k). Collectively, these findings indicate that, after a focal white matter lesion, grey matter microglia detect metabolic changes in neuronal somata evoked by axonal demyelination.

## Grey matter microglia modulate neuronal activity

As enhanced microglial–neuron interactions attenuate neuronal activity^40,42^, we examined whether microglia mediate the reduction in neuronal activity observed after focal demyelinating lesions.

**Fig. 4:**
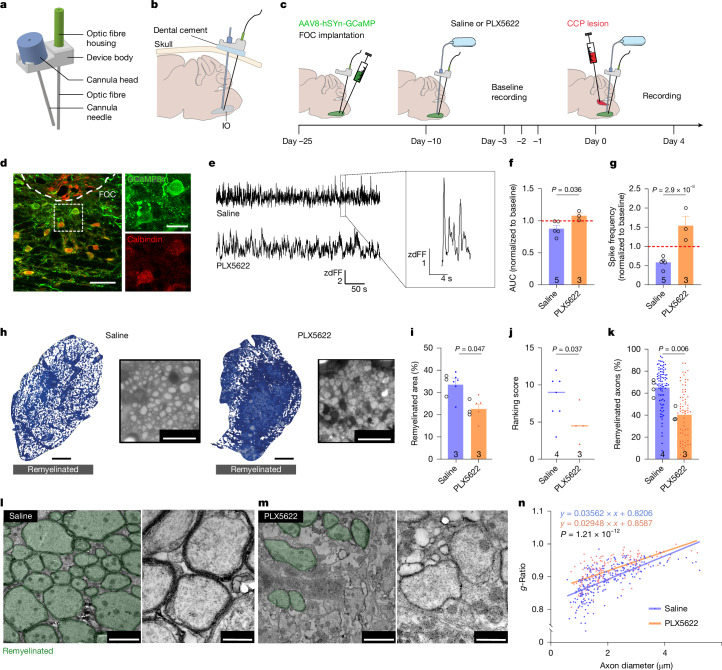
IO microglia regulate neuronal activity and white matter remyelination. **a**–**c**, Custom-made probe (**a**,**b**) and the experimental strategy for simultaneous activity recording and minipump-mediated delivery of PLX5622 (**c**). **d**, Representative (*n* = 5) immunostaining confirming GCaMP8m (green) expression in calbindin^+^ (red) neurons at the tip of the FOC. **e**–**g**, In vivo fibre photometry (*n* = 5 for saline and* n* = 3 for PLX5622) recording (**e**) and quantification of AUC (**f**) and spike frequency (**g**). **h**, Toluidine-Blue-stained semi-thin sections of the lesion. Remyelinated areas are displayed in white. Greyscale images show magnified areas. **i**, Automated quantification of the remyelinated area. **j**, Remyelination ranking. **k**–**m**, The percentage of remyelinated axons (**k**) quantified from EM micrographs (saline (**l**) and PLX5622 (**m**)). Remyelinated axons are pseudocoloured in green and magnified areas are shown in **l** and **m**. **n**, *g*-Ratios as a function of axonal diameter. The closed circles represent individual nested measurements: lesions (**i** and **j**) or axons (**k** and **n**) from *n* rats, indicated on the bars. The open circles show the rat means. In **n**, dots represent axons from *n* = 4 rats for saline and *n* = 3 for PLX5622. *P* values were calculated using two-sided *t*-tests (**f** and **g**), nested two-sided *t*-tests (**i** and **k**), two-sided permutation tests (**j**) and analysis of covariance (**n**). ANOVA *P* values are provided as Source data. Data are mean ± s.e.m., except for in **j**, in which the horizontal line represents the median. Scale bars, 1 μm (**l** (right), **m** (right)), 5 μm (**h** (high magnification), **l** (left),** m** (left)), 40 μm (**d** (right)), 100 μm (**d** (left)) and 200 μm (**h** (low magnification)). Source data

**Extended Data Fig. 7:**
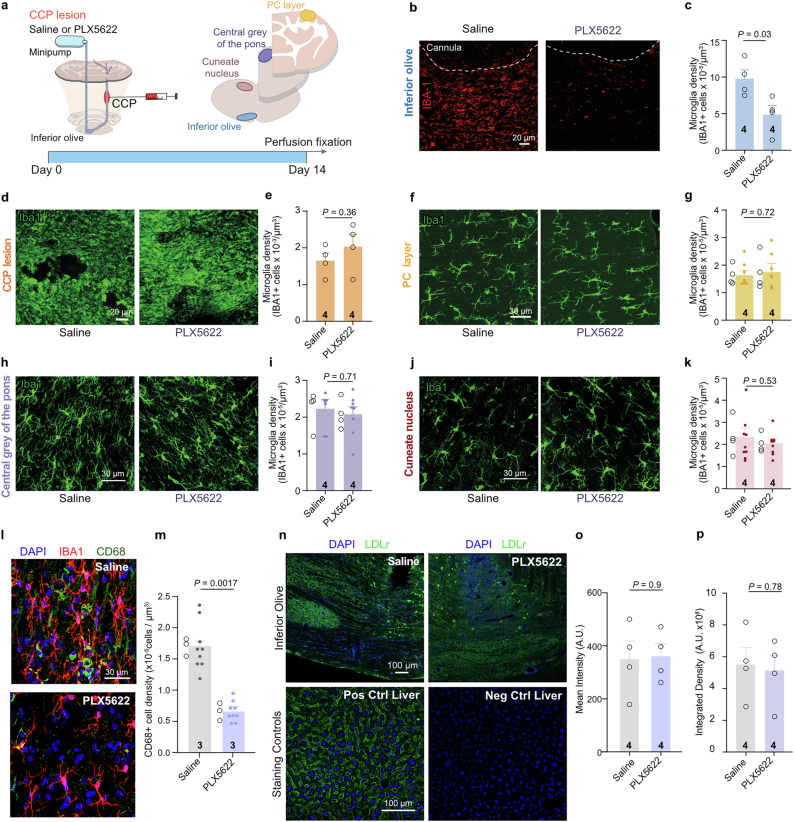
Focal PLX5622 delivery decreases local microglial density. **a**, Schematic representation of the local delivery of PLX5622 to the IO, and the different hindbrain regions analysed. **b**,**c**, IBA1 staining and quantification shows that microglia density is significantly decreased when PLX5622 is focally delivered to the IO. **d**,**e**, IBA1 staining and quantification shows that microglia density is unchanged in the CCP lesion. **f**,**g**, IBA1 staining and quantification shows that microglia density is unchanged in the PC layer. **h**,**i**, IBA1 staining and quantification shows that microglia density is unchanged in the central grey of the pons. **j**,**k**, IBA1 staining and quantification shows that microglia density is unchanged in the cuneate nucleus. **l**,**m**, Density of IBA1 (red) cells expressing CD68 (green), a microglial lysosome marker, is significantly reduced in the inferior olive of rats in which PLX5622 is locally delivered. **n**–**p**, Mean LDLr fluorescence intensity is not changed when PLX5622 is delivered to the IO (o). LDLr integrated density is not changed when PLX5622 is delivered to the IO. Images and quantification show that focal PLX5622 delivery does not affect LDLr expression, indicating that PLX5622-induced changes in the IO are not an off-target effect on cholesterol metabolism (p). Closed circles represent individual nested measurements from images (g, i, k, m) from N rats, indicated on the bars. Open circles**:** animal means. P values are from nested one-way ANOVA followed by Bonferroni t-test. Results are presented as mean ± s.e.m. P values are from two-sided t-test (c, e, o, p) or nested two-sided t-test (g, i, k, m). Results are given as mean ± s.e.m. Source data

**Extended Data Fig. 8:**
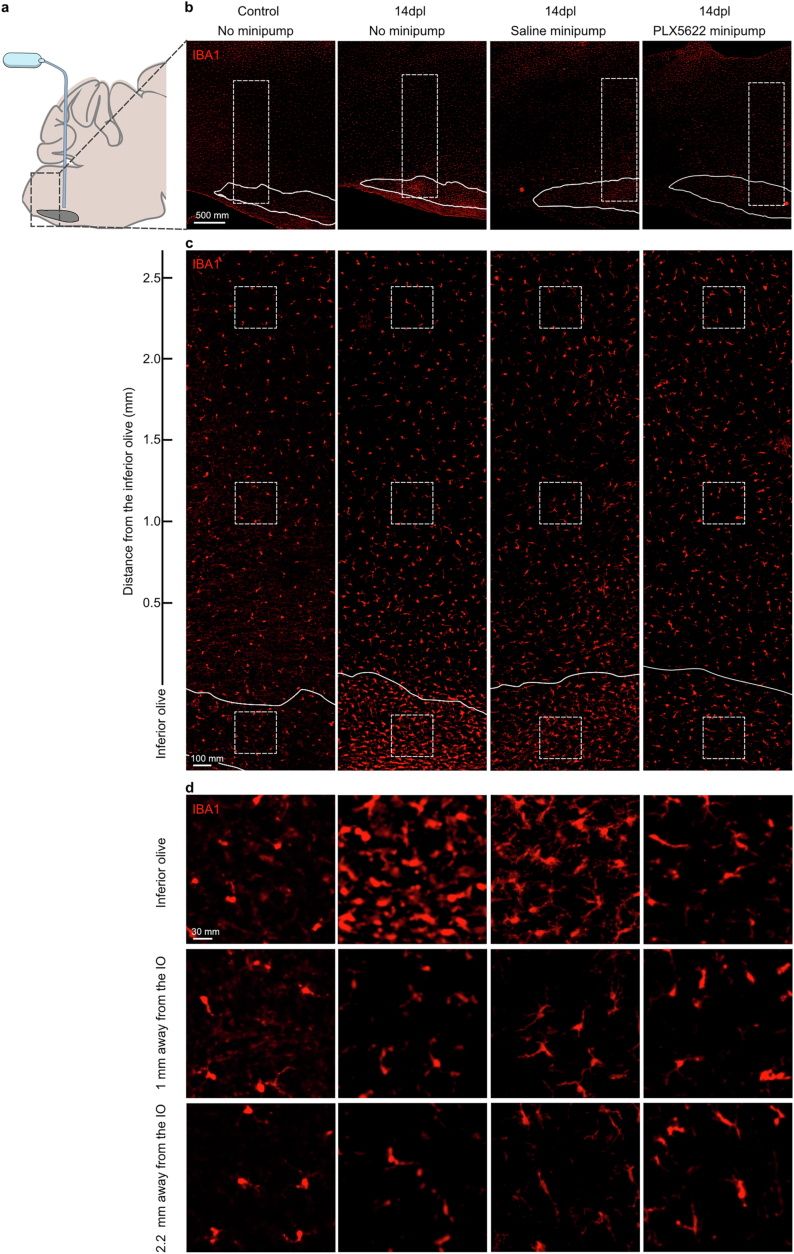
Detailed characterization of focal microglia depletion in the IO. **a**, Diagram showing the location of the inferior olive (grey) within the brainstem. **b**, Representative sections (N = 4) of the brainstem, containing the IO, stained for IBA1. **c**, Magnification of the area highlighted in **b**. The scale on the left indicates dorsal-ventral distance from the IO. **d**, Magnification of the boxed areas in **c**. Images show that 1) microglia density is increased at 14dpl in the IO, but not in areas > 1 mm dorsal to the IO and 2) that focal delivery of PLX5622 prevents such increase, with no effect on microglia density in areas above the IO.

We engineered a probe to record IO activity (GCaMP8m^+^calbindin^+^ neurons; AAV8-hSyn-GCaMP8m) while locally delivering PLX5622, a potent inhibitor of the microglial survival factor CSF1R^29^ (Fig. 4a–d). Local infusion of PLX5622 (10 µM, at 0.11 µl h^−1^) into the IO after a CCP lesion reduced the IO microglial density, quantified as IBA1^+^ or CD68^+^ cells (Extended Data Fig. 7a–c,l,m and Extended Data Fig. 8), without altering IBA1^+^ microglia/macrophages within the lesion (Extended Data Fig. 7d,e), in adjacent medullary regions or around CF–Purkinje-cell terminals (Extended Data Figs. 7f–k and 8).

After CCP demyelination, delivering saline into the IO replicated the transient reduction in neuronal activity observed previously (Figs. 1e,f and 4f,g). By contrast, neuronal activity remained unchanged when microglial CSF1Rs were inhibited with PLX5622 (Fig. 4e–g). Collectively these results indicate that grey matter microglia coordinate changes in neuronal activity and function after demyelination.

## Remyelination requires grey matter microgliosis

**Extended Data Fig. 9:**
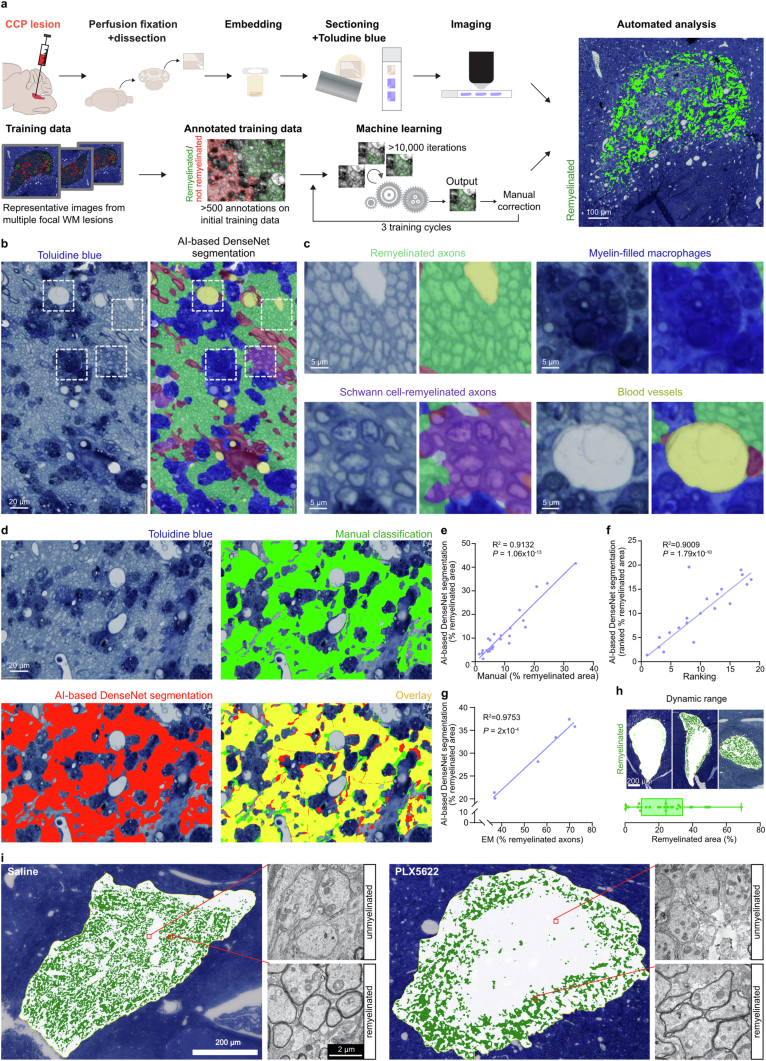
Detailed characterization of an AI-based DenseNet network for automated analysis of remyelination. **a**, Schematic showing the experimental design, tissue preparation and training of an AI-based Densenet network for automated analysis of remyelination. **b**, Representative (N = 20) toluidine blue-stained semi-thin section, imaged at the centre of a lesion (left) overlaid with the segmentation mask (right). **c**, Different objects segmented by the AI-based DenseNet network: remyelinated axons (green), myelin-filled macrophages (blue), Schwann cell remyelinated axons (purple) and blood vessels (yellow) (N = 20). **d**,**e**, Remyelinated areas measured by manual and automated segmentation are highly correlated (N = 20). **f**, Lesion ranking and ranked remyelinated area, measured by automated segmentation, are highly correlated (N = 20). **g**, Percentage of remyelinated axons, measured by EM, and remyelinated area, measured by the automated segmentation analysis, are highly correlated (N = 20). **h**, Dynamic range of the automated quantification. The trained network can discriminate between lesions with a wide range of remyelination, from lesions where remyelination has largely failed (0.3% of lesion area) to virtually fully remyelinated lesions (N = 20). **i**, Spatial correlation between automated segmentation and electron microscopy. Toluidine-blue sections were imaged at high magnification and images segmented using the AI-based DenseNet network. Consecutive sections were posteriorly ultrasectioned and corresponding FOVs imaged under the electron microscope. Images in the insets confirm that areas identified in the automated analysis as remyelinated and demyelinated contain primarily remyelinated and demyelinated axons, respectively (N = 1). Dots in graphs represent lesions. Curves were obtained using linear regression. P values are from an F-test to determine whether the slope of the curve is different from zero. Source data

The transience of these grey matter alterations and the fact that their resolution coincides with myelin regeneration suggests that microglial activation and synapse loss may not be detrimental but, rather, part of the myelin regenerative process. To test this, we induced a CCP focal lesion and delivered PLX5622 (10 µM, 0.11 µl h^−1^) through an intracerebral cannula into the IO. To determine the effect of suppressing the IO microglial response on CCP remyelination, we trained a DenseNet-based deep-learning image analysis pipeline (Halo-AI, Indica Labs; Methods and Extended Data Fig. 9a,b). The classifier was validated using correlative electron microscopy of adjacent sections and conventional ranking analysis by five independent assessors (Extended Data Fig. 9d–i).

Consistent with conventional ranking of semi-thin sections^21,43^, DenseNet-based deep learning analysis showed reduced remyelinated lesion area in PLX5622-treated rats (Fig. 4h–j). Ultrastructural analysis further showed fewer remyelinated axons (Fig. 4k), and slightly thinner myelin sheaths reflected by increased* g*-ratios (ratio between inner axonal area and total myelinated fibre area) (Fig. 4l–n). Collectively, this suggests that effective CCP regeneration requires an IO microglia response.

## The grey matter microglial response fails with ageing

**Fig. 5:**
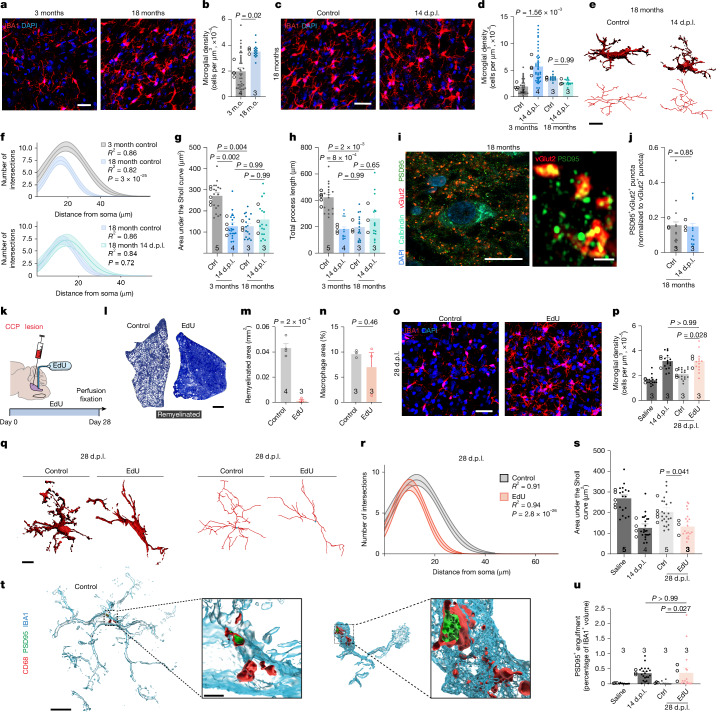
Failed white matter myelin regeneration leads to sustained microgliosis in the grey matter. **a**,**c**, IBA1 (red) immunostainings for 3-month-old (m.o.) (**a**) and 18-month-old (**c**) rats. **b**,**d**, Microglial density quantification for 3-month-old (**b**) and 18-month-old (**d**) rats. Microglial densities of 3-month-old control and 14 d.p.l. rats, also plotted in Extended Data Fig. 1i, are shown in **d** for comparison. **e**, Three-dimensional reconstructions and filament tracing. **f**, Gaussian fits of the Sholl analysis. **g**, Area under the Sholl curve. Data from 3-month-old control and 14 d.p.l. rats, also plotted in Fig. 3c, are shown for comparison. **h**, Microglial total process length. Data from 3-month-old control and 14 d.p.l. rats, also plotted in Extended Data Fig. 5a, are shown for comparison. **i**, Pre-synaptic (vGLUT2, red) and post-synaptic (PSD95, green) immunostaining. **j**, Synaptic punctum quantification. **k**, The experimental strategy for blocking remyelination. **l**, Toluidine-Blue-stained semi-thin sections of the lesion; remyelinated areas are displayed in white. **m**,**n**, Automated quantification of remyelinated (**m**) and macrophage (**n**) areas. **o**,**p**, IBA1 (red) immunostaining (*n* = 3) (**o**) and quantification (**p**). **q**, Three-dimensional reconstructions and filament tracing. **r**, Gaussian fits of the Sholl analysis. **s**, Area under the Sholl curve. Data from saline-treated and 14 d.p.l. rats are also displayed in Fig. 3c. **t**,**u**, Three-dimensional reconstruction (**t**) and quantification (**u**) of engulfed PSD95 (green). The closed circles represent individual nested measurements: images (**b**,**d**,**j** and **p**) or cells (**g**,**h**,**s** and **u**) from *n* rats, indicated on the bars. Open circles indicate the animal means. *P* values were calculated using nested two-sided *t*-tests (**b** and **j**), two-sided Wald *z*-tests with Holm correction (**f** and **r**), two-sided *t*-tests (**m**), Welch’s two-sided *t*-tests (**n**) and nested one-way ANOVA followed by Bonferroni post hoc comparisons (**d**,**g**,**h**,**p**,**s** and **u**). ANOVA *P* values are provided as Source data. Data are mean ± s.e.m. The middle solid line in the Gaussian fits indicates the mean, and the shaded area represents the 95% confidence interval. Scale bars, 1 μm (**i** (right)), 2 μm (**t** (high magnification)), 10 μm (**e**,**q**,**t** (low magnification)), 20 μm (**i** (left)), 30 μm (**a**,**o**), 40 μm (**c**) and 200 μm (**l**). Source data

To further validate the relevance of the grey matter microglial response to myelin regeneration, we investigated whether it is altered with ageing and associated regeneration failure^1,43^. In 18-month-old rats, the microglial density in the IO was higher than in 3-month-old animals (Fig. 5a,b), with no increase at 14 d.p.l. (Fig. 5c,d), and no lesion-induced morphological changes (Fig. 5e–h). Microglia in older animals, both lesioned and unlesioned, had reduced branching, shorter processes and enlarged irregular somata, resembling microglia at 14 d.p.l. in young rats (Figs. 3a–c and 5e–h and Extended Data Fig. 5a–d). Consistent with impaired microglial remodelling, old animals did not exhibit transient synapse loss as observed at 14 d.p.l. in young rats (Fig. 5i,j). This indicates that grey matter microglia accumulate with age, while becoming unresponsive to focal white matter demyelination. Given that transient increases in microglial density and morphological changes are necessary for myelin regeneration (Fig. 4h–n), these results provide an alternative explanation for why myelin regeneration fails in older animals.

## Failed remyelination sustains grey matter inflammation

**Extended Data Fig. 10:**
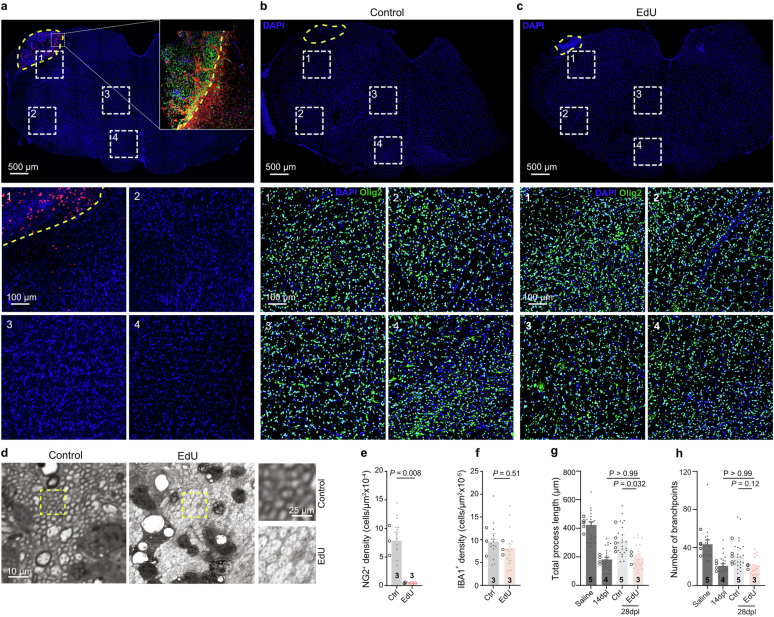
Detailed characterization of the chronic model of demyelination indicates that EdU delivery is focal. **a**, Representative (N = 6) images showing that EdU labelling is markedly restricted to the CCP lesion, indicating that EdU delivery is focal. The top image is a coronal section overview, stained for EdU (red) and DAPI (blue). The inset is a staining of the same lesion against GFAP (red) and IBA1 (green) and highlights the astrocytic scar around the lesion. Boxed areas are shown magnified below. The yellow dotted line outlines the lesion. **b**,**c**, Oligodendrocyte lineage cell (Olig2+) density is not different between control and EdU-infused rats (N = 9), indicating that the effect of EdU delivery is local to the lesion. The top images in **b** and **c** are coronal section overviews of control and EdU-infused rats. Magnified views of the boxed areas (bottom) show Olig2 staining. The yellow dotted line outlines the lesion. Lesions in EdU-infused rats (**c**) show high density of DAPI-labelled nuclei, which reflects ongoing inflammation in the absence of myelin regeneration. **d**, Representative (N = 4 for control and N = 3 for EdU) images showing that remyelination is impaired in rats in which EdU is locally delivered to the lesion. Images are from toluidine blue-stained sections of untreated (left) and EdU-infused lesions (right). **e**, NG2+ cell number is markedly decreased by EdU treatment in vitro. **f**, IBA1+ cell number is not affected by EdU treatment in vitro. **g**,**h**, Microglia total process length and mean branch number are significantly decreased in the IO when EdU is infused in the lesion. Data from saline and 14dpl are also displayed in Extended Data Fig. 5a,b. Closed circles represent individual nested measurements: images (e, f) or cells (g, h) from N rats, indicated on the bars. Open circles: animal means. P values are from nested two-sided t-test (e, f) or nested one-way ANOVA, followed by Bonferroni post-hoc comparisons (g, h). Results are presented as mean ± s.e.m. Source data

In progressive MS, failed myelin regeneration was previously considered to be the driving factor for disease progression and was attributed to intrinsic white matter changes, for example, impaired OPC recruitment and differentiation^1,14^. However, the emerging consensus suggests that progression is driven by widespread microglial-mediated grey matter neuroinflammation, independent of white matter lesions^5,7,18^. Our data show that demyelination evokes grey matter microgliosis and synapse loss in the IO, which resolves when myelin regeneration begins. We therefore predicted that failed myelin regeneration prevents the resolution of the grey matter microglial response, resulting in chronic microgliosis. To test this, we modelled regeneration failure by exploiting the sensitivity of OPCs to 5-ethynyl-2′-deoxyuridine (EdU). In vitro, 10 µM EdU blocked OPC proliferation and depleted NG2^+^ OPCs, without affecting IBA1^+^ microglia (Extended Data Fig. 10e,f). In vivo focal EdU delivery through a minipump into the CCP lesion resulted in myelin regeneration failure without affecting macrophages or myelin clearance (Fig. 5k–n and Extended Data Fig. 10a–d).

We next examined how preventing myelin regeneration in the CCP affected the IO microglia response. When myelin regeneration was prevented, all microglial parameters at 28 d.p.l. remained similar to the peak 14 d.p.l. responses rather than returning to homeostatic levels (Fig. 5o–s and Extended Data Fig. 10g,h). IBA1^+^ microglial density and 3D morphological analysis showed microglia remained in a phagocytic state (Fig. 5o–s and Extended Data Fig. 10g,h) with continued engulfment of excitatory synapses in the IO, comparable to 14 d.p.l. and indicative of continued synaptic pruning (Fig. 5t,u). These findings suggest that failed white matter regeneration shifts the grey matter microglial response from transient to chronic, implying that chronically demyelinated lesions may drive sustained grey matter inflammation and synapse loss, a phenotype that is common to multiple neurodegenerative disorders.

## Discussion

Here we used an anatomically defined circuit to demonstrate that focal white matter lesions evoke highly localized grey matter changes upstream of the lesion, around neuronal cell bodies of demyelinated axons, with little to no effect downstream. We demonstrate a causal link between focal white matter lesions and suppression of neuronal activity, grey matter microglial response and synapse loss. Grey matter microglia reduce neuronal activity and engulf synapses, and their response is required for white matter myelin regeneration. If microglia fail to respond, either due to depletion or age, myelin regeneration fails. Critically, failure of myelin regeneration leads to chronic microgliosis in the grey matter. This highlights the importance of considering the grey matter microglial response when designing myelin regenerative therapies. Our findings suggest that chronic neuroinflammation, implicated in MS progression and other age-related neurodegenerative disorders^7,11,12^, can be secondary to failed myelin regeneration, implying that myelin regenerative therapies could therefore be widely applicable across neurodegenerative disorders.

Focal white matter lesions increase with age and correlate with cognitive decline^1–3,13^ and occur early in disease alongside synapse loss^1–3,5,13,19,44^. Their consequences for circuit function have not been directly studied, and few studies have investigated the effect of demyelination beyond the lesion^5,44^. Animal models, human magnetic resonance imaging and post-mortem analyses show that white matter lesions and synapse loss coexist across neurodegenerative conditions, yet are considered unlinked. Global myelin dysfunction can accelerate and exacerbate pathological phenotypes in an Alzheimer’s disease model^17^, underscoring the important but underexplored role of white matter in neurodegeneration. Our study shows that a small focal demyelinating lesion, irrespective of its initial cause, is sufficient to initiate the cascade of events described here. Further investigation in appropriate disease models will be needed to establish whether similar mechanisms occur in neurodegenerative disease.

The anatomy of the olivocerebellar circuit has enabled us to untangle the spatiotemporal sequence of events after a white matter lesion. We find that these lesions precede microgliosis, synapse loss and altered neuronal activity, establishing them as potentially early events in neurodegenerative diseases. After demyelination, axonal conduction slows, delaying inputs to downstream terminals. Previous assumptions were that the response to a white matter lesion would occur at these terminals^5,6^ as microglia prune weaker synaptic connections during development^31^. Contrary to this, we show that the grey matter response to a focal white matter lesion occurs upstream in the circuit, surrounding demyelinated neurons, rather than at downstream synaptic terminals.

Our results highlight the importance of considering the entire circuit when interpreting post-mortem studies rather than focusing on isolated areas, as demyelinating injury along an axon is expected to affect cells surrounding its cell body, even when distant. The question remains whether all circuits respond similarly; however, we find comparable outcomes across multiple circuits.

The mechanism underlying remote microglial activation distant from focal white matter lesions is not entirely understood. Similar activation has been observed after peripheral nerve transection^26^ or a high salt diet^45^, indicating that localized gliosis represents a generic response to insult. However, unlike in these conditions, we do not detect GFAP upregulation in astrocytes, suggesting that this is a homeostatic response representing regenerative plasticity rather than a response associated with persistent or irreversible damage.

Our mitochondrial analysis and spatial transcriptomics data suggest the initial steps of regenerative plasticity involve mitochondrial remodelling, whereby mitochondria are shuttled down axons to meet energy demands at the demyelinated lesion, with increased mitochondrial fusion in the neuronal soma to enhance calcium buffering and ATP production.

Our data support a model in which demyelination triggers upregulation of mitochondrial and calcium-buffering genes as an immediate stress response. We propose that mitochondrial dynamics shift from ATP release at microglial–neuron contact points towards intracellular production to meet the increased neuronal metabolic demands after demyelination. Collectively, this remodelling reduces extracellular ATP release from neurons, which is probably sensed by microglia through P2Y12R at somatic purinergic junctions with demyelinated neurons^39^. Microglia respond by further reducing neuronal activity, thereby decreasing metabolic demands and providing neuroprotection during the demyelination period. Whether microglia promote the return of neuronal activity and synapse levels to base levels, as they do after anaesthesia^42^, needs to be further investigated, but such a mechanism could explain why white matter myelin regeneration depends on a microglial response in the grey matter, as microglia affect neuronal activity, and neuronal activity drives OPC differentiation and remyelination in the white matter^21,22^.

A role for grey matter microglia in promoting myelin regeneration parallels their emerging role regulating myelination during development^46^. Our findings show myelin regeneration in the white matter is critical to reduce synaptic engulfment and return microglia to a more homeostatic state, effectively switching off the grey matter inflammatory response. When myelin regeneration fails, the inflammatory response continues unabated and causes excessive synaptic engulfment. This failed regenerative state closely resembles the chronic low-grade neuroinflammation reported in neurodegenerative diseases and progressive MS.

This mechanism provides insights into why myelin regeneration fails with age, as in older animals microglia fail to respond to demyelinated lesions and already show activated-like morphology. This aligns with broader observations of declining or altered microglial functions during aging^12,47^, including baseline activation states and altered purinergic signalling that may impair their ability to sense and respond to neuronal ATP signals^29,48^. Recent studies also report accumulation of CD8^+^ T cells in the brain of older individuals, which can influence microglial activation and white matter integrity through interferon-γ signalling, although T cells in young CNS, including CD8^+^, can promote oligodendrocyte differentiation after demyelination^49,50^. The involvement of the adaptive immune system remains an important avenue for future research.

In summary, we identified a mechanism whereby a white matter demyelinating lesion induces a remote microglial response in the grey matter, which is required for white matter myelin regeneration. This response is transient but converts to sustained inflammation when myelin regeneration is impaired. This regenerative response represents a novel form of neuroplasticity—a neuroprotective mechanism that couples white matter integrity to grey matter function. Our data imply that myelin regenerative therapies could be critical not only to protect demyelinated axons and neurons, but also to alleviate chronic low-grade inflammation in MS and other neurodegenerative diseases.

## Methods

### Animals

Experiments were performed in accordance with the EU guidelines for the care and use of laboratory animals, and with the guidelines of the UK Animals (Scientific Procedures) Act 1986 and subsequent amendments. Use of animals in this project was approved by the Animal Welfare and Ethical Review Body for the University of Cambridge and carried out under the terms of UK Home Office Licenses PP4353554, P9B1FBC4B and 70/7715, and the Animal Welfare and Ethical Review Body by the Icelandic Food and Veterinary Authority. All animals were maintained under a 12 h–12 h light–dark cycle with food and water supplied ad libitum, in individually ventilated cages at 20–24 °C and 45–65% relative humidity. All surgeries were performed aseptically under isoflurane anaesthesia, with standard pre- and post-operative analgesia and care. Animals were randomized into experimental groups. Experimenters were not blinded to experimental conditions during animal surgery and recovery, as lesioned animals, but not unlesioned controls, can develop side effects that require enhanced monitoring. However, subsequent tissue processing, imaging and quantification were performed blindly and validated by more than two experimenters. Experiments that did not require animal surgery were performed blindly at all stages of data collection and quantification.

### Focal white matter demyelination in the CCP

#### EtBr model in rats

The CCP of 9–12-week-old female Sprague Dawley rats was bilaterally injected stereotaxically using a 10 μl Hamilton syringe. Four microlitres of EtBr (0.01% in saline) was injected at a rate of 1 μl min^−1^ to induce focal white matter demyelination. CCP coordinates were −7.3 mm (dorsal–ventral, DV); −2.3 mm (anterior–posterior, AP); ±2.6 mm (medium–lateral, ML) from lambda. In ageing experiments, CCP demyelinating lesions were induced in 18 month-old Sox10:dsRed rats.

#### EtBr model in mice

For mice, 3–5-month-old female C57Bl/6 mice were stereotaxically injected bilaterally with a mixture of EtBr and Dextran AlexaFluor 488 (Thermo Fisher Scientific; 1.50 μl, 0.01% and 2%, respectively, diluted in saline) at a rate of 0.25 μl per minute into the CCP; AP: −5.90 mm, ML: ±2.00 mm, DV −4.40 mm relative to bregma.

#### Lysolecithin model

Female Sprague Dawley rats (aged 9–12 weeks) were bilaterally injected with 2 µl of 1% lysolecithin (Sigma-Aldrich) in the CCP. Coordinates were AP: −2.3 mm; ML: ±2.6 mm, DV: −7.3 mm, relative to lambda.

#### Focal white matter hypoxic injury

Female Sprague Dawley rats (aged 9–12 weeks) were unilaterally injected in the CCP with 2 µl of 27 mg ml^−1^ L-NIO (N^5^-(1-iminoethyl)-L-ornithine) (Cayman Chemical), an irreversible inhibitor of nitric oxide synthase enzymes^30^. Two per cent Alexa-488 dextran was added to the injected mixture to retrogradely label IO neurons of which axons were exposed to L-NIO. Coordinates were as follows: AP, −2.3 mm; ML, ±2.6 mm; DV, −7.3 mm, relative to lambda. Rats were perfuse fixed at 14 d.p.l.

### Focal white matter lesion in the corpus callosum

Female C57Bl/6 mice (aged 6 months) were stereotaxically injected with 1 µl of lysolecithin (1%) into the corpus callosum. Coordinates were as follows: AP, −2.5 mm; ML, −0.8 mm; DV, −1.2 mm, relative to bregma. Mice were perfuse fixed at 14 d.p.l.

### Focal white matter demyelination in the cingulum

Female C57Bl/6 mice (aged 3–4 months) were stereotaxically injected with a mixture of EtBr and Dextran Alexa Fluor 488 (Thermo Fisher Scientific; 1 µl, 0.01% and 2% respectively, in saline) at a rate of 0.25 μl min^−1^, into the following cingulum coordinates: AP, +1.5 mm; ML, ±1.1 mm; DV, −2.1 mm, relative to bregma. Mice were perfuse fixed at 14 d.p.l.

### Chronic model of demyelination

To generate a model of failed myelin regeneration we capitalized on the knowledge that OPCs are highly proliferating progenitors, sensitive to EdU^51,52^. Thus, following focal white matter demyelination with injection of EtBr into the CCP of 9–12-week-old female Sprague Dawley rats, a 30-gauge cannula was implanted 100 µm above the lesioned area and connected to an osmotic minipump for local delivery of 10 µg µl^−1^ EdU (Sigma-Aldrich), as described previously^21^.

### Fibre photometry

#### Neuronal activity recordings

For neuronal activity recordings, the calcium indicator pGP-AAV-syn-jGCaMP7f-WPRE^53^ (1 μl, 5 × 10^12^ viral particles per ml; Addgene) or pGP-AAV-syn-jGCaMP8m-WPRE^54^ (1 μl, 5 × 10^12^ viral particles per ml; Addgene) were packaged into AAV8 particles and injected in the IO (DV, −10.6 mm; ML, ±0.55 mm, relative to lambda; and AP, −13 mm relative to bregma) to drive the expression of GCaMP7f or GCaMP8m in IO neurons. GCaMP8m has comparable temporal detection range as GCaMP7f but with greater signal to noise ratio^54^. During the same procedure, an FOC (200 µm in core diameter; numerical aperture, 0.37; Neurophotometrics) was implanted and secured on the skull surface using a pair of surgical screws (Plastic One) and dental cement (C&B Superbond). Fibre photometry was performed using a FP3002 console (Neurophotometrics). Ca^2+^-bound GCaMP7f/GCaMP8m was excited at 470 nm, (LED power set to 50 µW); fluorescence from Ca^2+^-unbound GCaMP7f/GCaMP8m was also recorded (excitation at the isosbestic point, 415 nm) to control for motion artefacts and correct for photobleaching.

#### Extracellular ATP measurements

For in vivo recordings of ATP release, 9–12-week-old female rats were unilaterally injected in the IO with 1 μl of AAV8 particles containing pAAV-hsyn-GRAB_ATP1.0_ (2.5 × 10^12^ viral particles per ml; Addgene) and pAAV-hSYn-mCherry (2.5 × 10^12^ viral particles per ml; Addgene). GRAB_ATP1.0_ is a modified human P2Y receptor with permutated enhanced GFP that allows for real-time sensing of extracellular ATP^55^. During the same surgery, an optical fibre cannula was implanted, as described for GCaMP recordings. Dual-colour fibre photometry was performed using the FP3002 console (Neurophotometrics), with GRAB_ATP1.0_ excited at 470 nm and mCherry excited at 560 nm. The mCherry fluorescence signal was used to correct for motion artefacts and photobleaching.

In recordings for both neuronal activity and ATP extracellular levels, the sessions were 10 min long, during which animals were allowed to freely explore a cage identical to their home cage. After data acquisition, baseline correction and signal standardization were performed and normalized d*F*/*F* was calculated in MATLAB (R2025a) using a published script^56^. In brief, fluorescence intensity values recorded from the 470 nm (Int470) and 415/560 nm (Int415/Int560) LEDs were subtracted from the respective intensity median and divided by the s.d. The standardized zInt415/560 signal was fitted to the zInt470 signal using non-negative robust linear regression. Parameters of the linear regression were then used to find fitted zInt415/560 values (fitzInt415) and zd*F*/*F* calculated using the equation zd*F*/*F* = zInt470 − fitzInt415/560 and plotted against recording time. GraphPad Prism v.10.5.0 was used for detecting peaks in activity traces (threshold set to 1–2 s.d.) and calculate the AUC. For ATP recordings, the average raw GRAB_ATP1.0_ fluorescence signal was plotted after normalization to the raw mCherry fluorescence signal.

#### Combined focal drug delivery and neuronal activity recordings

For simultaneous activity recording and microglial depletion, a custom-made device, conjugating optical fibre and drug-delivery cannula, was manufactured. Both components were positioned in the device such that the optic fibre could record data from the region of interest of drug delivery; the angle of offset between both the fibre and cannula being 15°. Devices were built using an Asiga MAX UV SLA 3D Printer with PrintoDent GR-10 UV curable (385 nm) biocompatible resin. Once printed, the devices were sonicated in isopropyl alcohol twice for 15 min, then exposed in a ultraviolet (UV) crosslinker for 15 min to ensure that the resin was fully cured. Devices were then subsequently assembled by inserting the optic fibre into place in the device and adhering it with Loctite 4305 LC, a UV curable cyanoacrylate resin. The cannula was not adhered before the experiment to enable correct placement during the surgical procedure.

### Immunofluorescence

Animals were transcardially perfused with heparinized PBS, pH 7.4 (PBS) followed by 4% paraformaldehyde (PFA) in PBS. Brains were dissected and post fixed in 4% PFA for 1 h at 4 **°**C with gentle shaking. The brains were then either processed into 100-μm-thick vibratome sections or prepared for cryosectioning by incubation in 30% sucrose for cryoprotection, embedded in OCT, then cut into 10-μm-thick cryosections. Vibratome sections were blocked at room temperature with 10% goat or donkey serum and 0.5% Triton X-100 in PBS for 4 h followed by an overnight incubation with primary antibodies at room temperature. The primary antibodies were diluted in PBS. Sections were then washed with PBS three times, 20 min per wash, and incubated overnight at 4 **°**C with the appropriate secondary antibodies diluted in PBS. The sections were washed twice with PBS, incubated with DAPI (1 µg ml^−1^) for 10 min, washed with PBS and mounted in Fluoromount G. Coverslips of cultured cells and cryosections had minor changes to the protocol. Blocking was performed using 10% goat or donkey serum and 0.5% Triton X-100 in PBS for 1 h at room temperature; overnight primary incubation was performed at 4 °C; and secondary antibody incubation was performed for 1 h at room temperature. Images were collected using the Leica SP8 confocal microscope and Leica application suite X (LASX) software. Large brainstem overview *z* stacks were taken using an Evident VS200 slide-scanning system running SILA (speckle illumination acquisition), with a ×20 objective and 2.36 μm *z* spacing.

Spectral confocal reflectance microscopy was performed as described previously^57^. In brief, images of 100-μm-thick brain slices were taken using 488 nm, 561 nm and 633 nm laser light and reflectance was detected ±5 nm around the respective excitation wavelength.

The following primary antibodies were used in this study: rabbit CD68 (Abcam, ab125212, 1:100), mouse calbindin (Abcam, ab82812, 1:100), rabbit calbindin (Abcam, ab108404, 1:200), rabbit C1q (Abcam, ab182451, 1:100), mouse NG2 (Abcam, ab50009, 1:200), rabbit COX-IV (Abcam, ab16056, 1:100), chicken anti-m-cherry (Abcam, ab205402, 1:500), chicken anti-GFP (Abcam, ab13970, 1:1000), rabbit GABAARγ1 (Abcam, ab238130, 1:100), mouse P62 (Abcam, ab56416, 1:100), chicken GFAP (Antibodies.com, A85307, 1:300), goat IBA1 (Antibodies.com, a82670, 1:100), chicken MAP2 (Abcam, ab5392, 1:2000), rabbit IBA1 (Wako, 019-19741, 1:500), mouse PSD95 (Merck Millipore, MAB1596, 1:100), guinea pig vGLUT2 (Merck Millipore, AB2251-I, 1:500), mouse bassoon (Abcam, ab82958, 1:100), goat LDLR (R&D Systems, AF2255, 1:100), rabbit Olig2 (Merck, AB9610, 1:300). Secondary antibodies (all used at 1:500 dilution) include goat anti-rabbit Alexa Fluor 488 (Thermo Fisher Scientific, A32731), goat anti-rabbit Alexa Fluor 568 (Thermo Fisher Scientific, A11036), goat anti-rabbit Alexa Fluor 647 (Thermo Fisher Scientific, A21247), goat anti-mouse Alexa Fluor 750 (Thermo Fisher Scientific, A21037), goat anti-mouse Alexa Fluor 647 (Thermo Fisher Scientific, A21242), goat anti-mouse Alexa Fluor 555 (Thermo Fisher Scientific, A21424), goat anti-mouse Alexa Fluor 488 (Thermo Fisher Scientific, A11029), goat anti-mouse Alexa Fluor 568 (Thermo Fisher Scientific, A11031), donkey anti-goat Alexa Fluor 633 (Thermo Fisher Scientific, A21082), donkey anti-Rabbit Alexa Fluor 488 (Thermo Fisher Scientific, A-21206), goat anti-hamster Alexa Fluor 488 (Abcam, ab173003), goat anti-guinea pig Alexa Fluor 647 (Abcam, ab150187) and goat anti-chicken Alexa Fluor 568 (Abcam, ab175477). For microglial densities, CD68 expression, neuron counting, OPC counting and area measurement analysis, imaging used a ×20 objective with ×2 digital zoom, and *z* stacks were set up with 10 optical sections and 2.5 µm step size. Quantification of total number of IBA1^+^ microglia and the fraction of microglia expressing CD68 was performed in a blinded manner in ImageJ (v.1.54p). Areas containing the surgical needle track were excluded from the analysis. For analysis of synaptic densities, Zeiss LSM 980 with a Airyscan 2 module and Zen blue software (v.3.7.97.0.7000) were used. Imaging used the super-resolution mode (×2 sampling), with a ×63 objective and ×2 digital zoom, with *z* stacks set up to contain ten optical sections, with a 120 nm step size. Quantification of synaptic puncta density was performed in a blinded manner in ImageJ, using SynapseJ^58^. In all of the experiments, the laser power was adjusted to achieve a full dynamic range of intensity values.

### Microglial morphology and engulfment of synaptic material

One-hundred-micrometre thick vibratome sections were immunostained for IBA1, CD68 and PSD95 or bassoon. For each animal, three to five regions of interest within the IO were imaged using the Leica SP8 confocal microscope and Leica application suite X (LASX) software. Images were obtained using a ×63 oil immersion objective (NA 1.4) with a ×2 optical zoom and 0.3 μm optical section thickness. The pixel resolution was 1,024 × 1,024 and the voxel size was 0.090 × 0.090 × 0.297 μm^3^. The field of view was 92.4 × 92.4 μm^2^ and each stack contained 33–50 planes (10–15 μm). Raw images were processed on Imaris (Bitplane) v.9.1 and v.10.2 for analysis. Imaris Filament Tracer (Autopath) was used to reconstruct cell morphology and quantify total cell process length, number of branch points and number of Sholl intersections. The estimated largest diameter of soma to calculate starting points was 6–8 μm. To calculate seed points, the thinnest and largest diameters of microglia processes were 0.2 μm and 2.00 μm, respectively. Starting point and seed point thresholds were adjusted accordingly. Cell body roundness was quantified in FIJI (v.1.54p). Microglia cells were chosen at random for analysis. Autothreshold of the IBA1 channel was performed to saturate the outline of the cell body. The long and short axes of the cell bodies were measured (in μm). For each cell, the short axis was divided by the long axis to obtain an index measure of roundness from 0–1; a score of one corresponds to perfect roundness, and a score of zero corresponds to the opposite. Roundness index = short axis (μm)/long axis (μm). Soma size was quantified in the commercially available cloud-based deep learning platform (Aiforia Create, Aiforia Technologies, https://www.aiforia.com/). An algorithm was trained using a supervised learning approach to recognize and trace the two-dimensional outline of microglial cell bodies. Six hundred diverse and representative microglial cell bodies across all groups were manually annotated and cycled through 19 iterative training sessions to create a generalizable model. The algorithm was validated by analysis of untrained images.

### Microglial engulfment analysis

Imaging proceeded as for morphological analysis, with cells randomly selected for analysis. Imaris 3D surface rendering was used to analyse engulfment. First, a surface was created for the IBA1 channel. The CD68 channel was then masked to the IBA1 surface to obtain CD68 fully contained within IBA1 cells. A surface was then rendered for the masked CD68 channel. A mask was applied within CD68^+^ reconstructed lysosomes for PSD95 channel to obtain synaptic proteins inside the CD68^+^ lysosomes. Surfaces were created for the masked PSD95 channel. Data were calculated and presented as an engulfment index: (volume PSD95 within CD68+ lysosomes/IBA1 volume) × 100.

### Analysis of microglial–neuron interactions

Vibratome sections (thickness, 100 µm) were stained for IBA1 and calbindin. Images of the IO were obtained using a ×20 objective with a ×2 optical zoom and 2.3 μm optical section thickness. Pixel resolution was 1,024 × 1,024 and voxel size 0.284 × 0.284 × 2 μm^3^. The field of view was 291 × 291 μm^2^ and each stack contained 5 planes (10 μm). Maximum-intensity projections of full *z* stacks were generated in ImageJ and all IBA1^+^ microglia in the field of view were categorized as interacting or non-interacting with calbindin^+^ neurons. Interacting microglia comprised microglia extending processes that contacted neurons, microglia with cell bodies in juxtaposition to neuronal cell bodies and microglia in which cell bodies and processes enwrapped neuronal cell bodies^59^. The density of interacting microglia was obtained by dividing absolute numbers of interacting microglia by the volume of the *z* stack.

### Tissue culture

Cells were maintained at 37 °C and 5% CO_2_. Primary mixed glial cultures, OPCs, and microglia were prepared as described previously^60^. OPCs were resuspended in 50% DMEM, 50% Neurobasal, with modified SATO, 10 ng ml^−1^ biotin, 2% B27 and 1× GlutaMAX (Invitrogen), and 1 × 10^5^ OPCs were plated per well onto 12-mm-diameter PDL-coated glass coverslips in a 24-well plate, with daily addition of growth factors (PDGF-aa at 10 ng ml^−1^ and FGF-b at 10 ng ml^−1^; Peprotech). Microglia were plated at 1 × 10^4^ cells per well and cultured in DMEM containing 10% FCS (Life Technologies) and 1% penicillin—streptomycin; after 24 h, half of the medium was changed to the same culture medium as used for the OPCs. EdU (10 µM) was applied to the OPC and microglial cultures the day after plating; cells were fixed in 4% PFA 48 h after EdU application.

### Fast Blue labelling and flow cytometry

The CCP was lesioned and, in the same procedure, injected with 0.5 µl of 0.5% Fast Blue for retrograde labelling of demyelinated neurons in the IO and associated microglia. Then, 3, 7, 14 and 28 days after lesion, rats were decapitated, and microdissected IO tissue was digested with papain solution in DMEM buffer for 30–45 min at 37 °C. Cells were triturated to single cells after blocking papain activity with ice-cold soybean trypsin inhibitor solution, then passed through a 40 µm strainer. Cells were separated from myelin debris using the myelin removal microbead kit from Miltenyi (130-096-733) according to the instructions. Dead and dying cells were labelled with propidium iodide for 5 min in PBS without Ca^2+^ and Mg^2+^ supplemented with 0.5% BSA fraction V before analysis. Cells from control cortical tissue were heat-shocked at 45 °C for 2 min to provide a positive control. The samples were analysed on the Beckton Dickinson FACS Aria2 system using FACSDiva (v.9.0.1) software. Single-cell gating and Fast-Blue-negative and propidium-iodide-negative populations were defined using cortical cells and heat-shocked cortical cells.

### Spatial DBiT-seq

Spatial transcriptomic sequencing was performed according to the previously published protocol and reagents^61^. In brief, 76 × 52 mm slides with four mounted lesion and IO sample sets (8 coronal sections; see the diagram in Fig. 2a) were thawed at room temperature for 10 min then fixed in 0.2% formaldehyde (−methanol) for 5 min at room temperature. The fixation was quenched with 1.25 M glycine for 5 min.

In situ reverse transcription was performed by adding reverse transcription mix in a humidifying chamber, the tissue was first incubated at room temperature for 30 min, then at 42 °C for 90 min. The samples were washed with 1× NEB Buffer 3.1 plus RNase Inhibitor and bright-field imaging was performed using the Zeiss Cell Observer system.

In situ spatial ligation barcoding was completed by first loading barcode A ligation reactions (100; Fig. 2a) into the vertical microfluidic PDMS chip followed by the second round of barcode B ligation reactions (100; Fig. 2a), which were run on a horizontal 5 pass PDMS chip. In situ barcoding with the ligation mix for each round of barcoding was prepared and a total of 4 μl of ligation mix was added to each 1 µl barcode + linker mix (A001-A100 or B001-B100) with additional loading of Alexa Fluor 488 dye and 647 dye in position 1 and 100 to demarcate the region of interest borders. The cassette, PDMS chip and slide were assembled so that the fluidic lanes were bypassing each region of interest, then 4.5 µl of the barcoded ligation mix was added to each microfluidic lane on the input side of the chip. A vacuum gasket was set over the outlet wells, and 200–300 p.s.i. of suction pressure was applied for 7 to 10 min. Lane-loaded barcoded ligations with the cassette intact were incubated for 30 min at 37 °C in a humidifying chamber. Cassette disassembly and sample washing was done before in situ B barcoding (following the same steps as for DAPI staining and bright-field and fluorescence imaging using the Zeiss Cell Observer system).

Reverse crosslinking of each region of interest was separately processed by first assembling a PDMS chip (with five regions of interest) cassette on each slide. Reverse crosslinking buffer was added to each well, then incubated at 58 °C for 2 h. Each lysate was transferred to an individual tube then further incubated overnight at 65 °C with agitation.

The cDNA library was constructed by first performing streptavidin-bead affinity pull-down then template switching using template-switch oligo mix that was added to each bead sample. The samples were incubated for 30 min at room temperature then for 90 min at 42 °C. The beads were washed then resuspended in PCR solution. Thermocycling was done (stage 1, 1 cycle, 95 °C for 3 min; stage 2, 5 cycles, 98 °C for 20 s, 65 °C for 45 s, 72 °C for 3 min; stage 3, 1 cycle, 72 °C for 1 min, 4 °C hold). The supernatant was removed from the streptavidin beads and a quantitative PCR (the same thermocycling programme except the 65 °C cycle was 20 s instead of 45 s) assessment to determine the final cycle number to achieve 1/3 of saturation signal was completed. Additional cycles were run on the remaining sample according to quantitative PCR results. Then SPRI bead cleanup was performed, and samples were eluted in EB buffer.

To prepare the final library for sequencing, the mRNA-derived cDNA was quantified using Qubit and BioAnalyzer assessment. Depending on results further purification was performed as needed. Nextera XT Library Prep Kit was used and the Tagment Mix was incubated at 55 °C for 5 min. NT buffer was added and the sample was then incubated at room temperature for 5 min. The final PCR mix was added and thermocycling was completed (stage 1, 1 cycle, 95 °C for 30 s; stage 2, 12 cycles, 95 °C for 10 s, 55 °C for 30 s, 72 °C for 30 s; Stage 3, 1 cycle, 72 °C for 4 min, 4 °C hold). The concentration and size distribution were determined using the Agilent Bioanalyzer High Sensitivity Chip. Next-generation sequencing was completed at NGI (National Genomics Infrastructure) using Illumina NovaSeq X Plus 25B in paired-end 150 bp mode with a total of 6 million reads for each sample and a 15% PhiX sequencing step.

Samples at 28 d.p.l. did not pass quality control and were excluded from all analysis.

### DBiT-seq bioinformatics analysis

Tissue images acquired using the Zeiss Cell Observer microscope were imported into R (v.4.3.3) using the imager package (v.1.0.2)^62^. PDMS chip imprints were manually identified in each image and extracted. Extracted regions were converted to greyscale, and morphological operations (pixel erosion and dilation) were applied to reduce imaging artefacts while preserving tissue structure. To define the cassette pixel layout, the dimensions of the extracted tissue image were divided by the number of channels in the cassette, generating a grid corresponding to cassette channels positions. For each cassette pixel location, the number of underlying image pixels was aggregated to produce a pixel-intensity distribution mapped to the cassette structure. To correct for imaging artefacts—particularly in regions lacking tissue or along cassette borders—cassette pixel values were adjusted using a combination of internal pixel intensities, neighbouring pixel values and proximity to the chip edges. Regions with obvious absent tissue were identified and used as a distribution threshold to exclude off-tissue grid coordinates. The final set of valid cassette coordinates was exported as a tab-delimited file for downstream analyses.

A total of 15 transcriptomes was sequenced at the in-house sequencing facility. Spatial barcode demultiplexing and FASTQ file preparation were performed using Snakemake (v.7.24.0)^63^ as the workflow management system. Sequencing reads from multiple lanes were merged into single FASTQ files per sample. Reads were filtered out using bbduk (v.39.01)^64^, if either PCR primers or spatial linkers were missing from the read construct. Spatial barcodes were extracted from sequencing reads using a custom Python script and written to separate FASTQ files. A predefined whitelist of spatial barcodes was supplied—annotation following the in situ spatial ligation barcoding—and cellranger count (v.7.1.0) was used to align reads to the *Rattus norvegicus* reference genome (mRatBN7.2, RefSeq annotation, NCBI). Tissue images, spatial barcode coordinates and gene count matrices were imported into R. Count matrices were loaded into a Seurat object (v.5.1.0)^65^, retaining only barcodes corresponding to tissue-covered pixels. All of the samples were subsequently merged into a single expression matrix for downstream analysis.

To account for potential lane clogging inherent to the DBiT-seq technology, which can result in abnormally low transcript counts in affected lanes, we implemented a filtering and imputation strategy. Vertical and horizontal lanes exhibiting total gene counts substantially below the distribution observed across the full dataset were identified as outliers and set to zero. Subsequently, gene expression values in these lanes were imputed by averaging the expression values of their immediately adjacent lanes. This correction was applied solely for purposes of clustering and visualization to improve spatial coherence. All DGE analyses were performed on the original, uncorrected expression matrix.

Gene count matrices were normalized by total library size, scaled by a factor of 10,000 and natural-log-transformed. Genes were then standardized by centring their mean expression and dividing by their s.d. Dimensionality reduction was performed by principal component analysis (PCA) on the 2,000 most variable genes, selected using the vst method. The first 30 principal components (PCs), determined by elbow plot inspection, were retained for downstream analysis. Samples were integrated using the IntegrateLayers function in Seurat, applying the reciprocal PCA (RPCA) integration method.

Neighbour graphs were constructed from the selected PCs using the FindNeighbors function with the ‘annoy’ approximate nearest neighbour algorithm, Euclidean distance metric and 50 trees. The number of nearest neighbours (*k*) was set to 20. Unsupervised clustering was performed using the FindClusters function with the Louvain algorithm and a resolution parameter of 1.2. For visualization, uniform manifold approximation and projection (UMAP) was computed from the first two dimensions of the neighbour graph.

IO gene expression markers were used to delineate an IO-specific pixel cluster from whole-tissue datasets. For each tissue, a *k*-nearest neighbour (*k*-NN) graph (*k* = 5) was constructed using the get.knn function from the FNN package (v.1.1.4.1)^66^ and subsequently converted into a graph object with graph_from_edgelist from the igraph package (v.2.1.4)^67^. Clusters within IO-associated pixels were identified using the components function. The largest cluster was defined as the principal IO cluster, with additional clusters of at least half the main cluster size to be aggregated to the main cluster. To exclude peripheral pixels from the main cluster, we applied a 0.5 quantile threshold on average graph edge distances per node. The coordinates of pixels within the main cluster were then dilated by a radius of two pixels in all directions to generate a contiguous IO region of interest.

Lesion sites were delineated manually, guided by staining of IBA1 immunohistochemistry on adjacent sections delineating the lesion location, along similar anatomical landmarks. Control regions were defined as 10 × 10 pixel squares on the ipsilateral side, spatially separated from the lesion.

To characterize calbindin-expressing neurons within the IO, all *Calb1*^+^ pixels were selected for subsequent analyses (total 3,442 pixels out of total 10,864 pixels allocated to the IO region). To characterize microglia within the lesion, a microglia score was computed using either three (*P2y12r*, *Tmem119* and *Cx3cr1* identified 1,289 pixels) or four marker genes (*Aif1*,* Maf*,* Spi1* and *Csf1r*; 2,874 pixels identified) with the AddModuleScore function from Seurat, specifying ctrl = 100 and nbin = 24. Both approaches gave similar results. To characterize microglia within the IO, a microglial score was computed using four marker genes (*Aif1*,* Maf*,* Spi1* and *Csf1r*) using the AddModuleScore function from Seurat, specifying ctrl = 100 and nbin = 24. Pixels with a microglia score greater than 0.05 were extracted from downstream analyses.

Gene count matrices were normalized to total library size, scaled by a factor of 10,000 and log-transformed (natural log). Gene expression values were then standardized by centring on the mean and scaling by the s.d. Dimensionality reduction was performed using PCA on the 1,000 most variable genes, identified using the vst method. The number of retained PCs—20 for IO, 20 for lesion, 20 for IO calbindin neurons and 15 for IO microglia—was determined by elbow plot inspection. Neighbour graphs were constructed from the selected PCs using FindNeighbors with the annoy approximate nearest-neighbour algorithm, Euclidean distance metric and 50 trees, with *k* set to 20. Unsupervised clustering was carried out using FindClusters (Louvain algorithm) at resolution parameters of 1 (IO), 0.5 (lesion), 0.8 (IO calbindin neurons) and 0.7 (IO microglia). For visualization, UMAP was computed from the first two dimensions of the neighbour graph. The number of pixels in the top levels of the chord/circos plots corresponding to each bottom-level category was quantified. To enable an unbiased visualization of proportional relationships, top-level categories were downsampled before plotting.

### Bulk RNA-seq libraries

After the flow sorting above, Fast Blue^+^propidium iodide^−^ cells were isolated directly using a 100 µm nozzle into 900 µl volume of RLT buffer of Qiagen RNeasy micro kit without β-mercaptoethanol. The isolated cell lysate was kept at 4 °C during the isolation process. Total RNA was isolated using the Qiagen RNeasy micro kit and kept at −80 °C until use. The RNA integrity number (RIN) was calculated for each sample for quality control, with a mean RIN of 9.57. A mRNA library was prepared using SMARTer Stranded Total RNA-Seq Kit v3 - Pico Input Mammalian (Takara Clontech) according to manufacturer’s instructions. Data were read on the HiSeq4000 system; a total of >350 million 150 bp strand-specific paired-end reads were generated.

### Bulk RNA-seq deconvolution and bioinformatics analysis

*R. norvegicus* (Rn7) bulk RNA-seq raw count matrixes were converted to *Mus musculus* (Mm10) using the EnsEmbl Biomart database. Only orthologue genes with a conservation score of at least 80 between mm10 and Rnor7, ENSEMBL v.102, were selected for downstream analyses. The final number of genes was reduced from 33,295 genes to 15,678 orthologue genes.

Deconvolution was performed in R v.4.5.0 environment. For the single-cell RNA-seq reference, the medulla matrix from ref. ^68^ was downloaded (https://cells.ucsc.edu/?ds=mouse-nervous-system)^69^ and converted to SingleCellExperiment^70^ R object with SubClass and SampleID metadata labels. The raw count matrix with mouse annotations and ref. ^68^ medulla data were used as an input for MuSiC R package^71,72^. MuSiC was run with function music_prop for neurons and microglia (labelled as immune previously^68^) using the sample IDs from ref. ^68^. For each gene, the mean expression in each sample was first calculated. Then, based on the microglial and neurons deconvoluted proportions from MuSiC an inferred relative gene proportion was calculated. The individual gene inferred proportions were then rounded and converted to inferred counts. This measure is an approximation and is dependent on the proportions predicted from MuSiC.

The expression of the top 200 fold-change (versus 28 d.p.l.) genes is shown as heat maps in Fig. 3d. Heat maps were plotted using ComplexHeatmap R package^73^ (ComplexHeatmap (Bioconductor; http://bioconductor.org/packages/ComplexHeatmap/). Final visualization shows the scaled *z* scores of the top 200 fold-change (versus 28 d.p.l.) genest (scale function center = TRUE). GO term analysis was performed using EnrichR v.3.4 package^74^.

### Analysis of mitochondrial density and morphology

Female rats (aged 9–12 weeks) were injected with 1 µl of pAAV2-CAG-MitoDsRed-IRES-GFP (a gift from J. Kittler) to genetically label mitochondria in the IO. The coordinates for stereotaxic injections were as follows: DV, ±10.6 mm; ML, ±0.55 mm relative to lambda; and AP, −13 mm relative to bregma. Then, 3 weeks after viral injection, the CCP was lesioned or injected with saline and rats perfused at 3 d.p.l. Then, 100-µm-thick parasagittal sections were produced with a vibratome and stained for IBA1 and calbindin.

For analysis of mitochondria in calbindin^+^ neuronal cell bodies, images were taken on a Leica SP8 confocal microscope with a ×63 objective, ×3 digital zoom and pinhole at 0.35 AU, and *z* stacks were taken with a 0.15-μm step size to image the entire cell. Images were deconvolved using Huygens Professional, following default settings and using 20–30 iterations. Deconvolved images were imported into FIJI and analysed in three dimensions using the Mitochondria Analyzer plugin, as previously described^75^. Thresholding and image processing were used according to the plugin workflow. Analyses were performed at both the per-cell and per-mitochondrion levels. Per-cell analysis was defined as the mean measurement of all mitochondria within a cell body; per-mitochondrion analysis represented measurements of each individual mitochondrion measured. The following parameters were extracted: mitochondrial density (mitochondrial count normalized to cell body volume in µm^3^), mitochondrial volume (summed volume of all mitochondria normalized to cell body volume) and volume-weighted mitochondrial sphericity (sphericity weighted by each mitochondrion’s contribution to total mitochondrial volume). Values closer to 1 indicate more spherical morphology. The formulas used for these calculations were adapted from the Mitochondria Analyzer documentation. If *S*_*i*_ is the sphericity of mitochondrion* i*, *V*_*i*_ is its volume and SA_*i*_ is its surface area:

$${S}_{i}=\frac{{{\rm{\pi }}}^{1/3}\times {(6\times {V}_{i})}^{2/3}}{{\mathrm{SA}}_{i}}$$

$${\text{Average Cell}\, S}_{\mathrm{weighted}}=\frac{{\sum }_{i=1}^{n}{V}_{i}\times {S}_{i}}{{\sum }_{i=1}^{n}{V}_{i}}$$

Cell body volume was estimated from the longest *x* and *y* axes of the soma together with the *z*-stack depth, using an ellipsoid volume approximation:

$${V}_{\mathrm{cell}}=\frac{4}{3}{\rm{\pi }}\times \frac{x}{2}\times \frac{y}{2}\times \frac{z}{2}$$

For analysis of mitochondria in neuron–microglia junctions, images were taken with a Leica Stellaris confocal microscope with ×63 objective, ×4 digital zoom and pinhole at 0.35 AU and *z* stacks were taken with 0.15 μm step size. Images were deconvolved using Huygens Professional, using the default settings and 20–30 iterations. Mitochondria in neuron–microglia junctions were analysed using Imaris 10.2 (Bitplane). In brief, surfaces were generated from both the IBA1 and the MitoDsRed channels, slightly exaggerated to highlight the overlap between volumes. These surfaces were then used to mask the individual channels, and the masked channel was used to create faithful surfaces for IBA1 and MitoDsRed. Segmented mitochondria statistics were exported from Imaris for downstream analysis.

### Local microglial depletion

Female rats (aged 9–12 weeks) were stereotaxically injected with EtBr (4 µl, 0.01% in saline) to induce a demyelinating lesion in the CCP. During the same procedure, a 30-gauge cannula (Plastics One) was implanted into the IO and connected to an osmotic minipump (Alzet Micro-Osmotic Pumps, model 1004, DURECT), with a flow rate of 0.11 μl h^−1^. PLX5622 (10 μM; MedChemExpress), a potent Csf1r blocker, was continuously delivered to the IO to locally deplete microglia.

### Histological analysis of remyelination

For analysis of remyelination levels in the lesion, rats were perfused with 4% glutaraldehyde in phosphate buffer, followed by 5 days of post-fixation. One-mm-thick sections containing the lesion were further fixed in 2% osmium tetroxide, overnight, at 4 **°**C; washed three times in PBS; dehydrated using 70%, 95% and 100% ethanol, followed by 100% propylene oxide and embedded in resin. Blocks of resin-embedded tissue were sectioned (1 μm), stained with 1% toluidine blue and imaged on a Zeiss Axioscan (×40 objective). The remyelinated area was quantified using Halo-AI v.3.6.4134 (Indica labs). Image analysis pipeline was generated by training an AI-based DenseNet network to automatically quantify the remyelinated area within focal white matter lesions at 28 d.p.l. The network was trained to segment the lesion area into remyelinated and non-remyelinated regions. More than 500 annotations on 10 representative lesions were produced and the network was trained for over 10,000 iterations with manual correction cycles until achieving excellent classification accuracy (cross-entropy < 0.001). The classifier was validated using correlative electron microscopy analysis of adjacent sections and conventional ranking analysis conducted by five independent assessors (Extended Data Fig. 9). Analysis of remyelination by the automated segmentation, ranking and electron microscopy was highly correlated. Remyelination by Schwann cells was excluded in the analysis. For each analysis automated segmentation was inspected, if needed classified tissue areas manually corrected and the network was further trained for a total of three cycles. Remyelination was further assessed by ranking, performed blindly and independently by two scorers and by electron microscopy.

### Electron microscopy

Toluidine-Blue-stained semithin sections used for automated histological analysis of remyelination were glued to empty resin blocks and repeatedly dipped into liquid nitrogen until they detached from the glass slide. Ultrathin sections were cut at 75 nm (Leica EM UC7 ultramicrotome, DiATOME Ultra 35° knife), transferred to copper grids and post-stained for 1 min with UA-Zero and 30 s with lead citrate. The lesion was identified in 4 × 4 0.5kx overview images, then ten images were taken at random lesion positions per animal (Hitachi HT7800 TEM, 80 kV, 3kx magnification, EMSIS Xarosa camera, 5,120 × 3,840 px, 4.97 nm per px). Quantification of the percentage of remyelinated axons and *g*-ratios were performed in ImageJ, remyelination by Schwann cells was not included in analysis.

### Quantification and statistical analysis

All statistical analyses were performed in GraphPad Prism v.10.2.3 and v.10.5.0 or in Python (v.3.11.13). Data are shown as mean ± s.e.m., with the number of animals (that is, the biological replicates) indicated in the figures and Supplementary Table 1. For non-hierarchical data (single measurement per animal), two-tailed Student’s* t*-tests (with Welch’s correction when variances were unequal) were used for two-group comparisons. For more than two groups, one-way ANOVA was used (Welch’s ANOVA when variances were unequal), followed in all cases by Bonferroni post hoc comparisons to correct for multiple comparisons. Consistent with statistical best practises in neuroscience^76–79^, for hierarchical data (for example, cells or images nested within animals), nested analyses were applied to preserve within-animal variability. Two-group comparisons used nested two-tailed *t*-tests, and multiple-group comparisons used nested one-way ANOVA, followed by Bonferroni post-hoc comparisons. Where assumptions of normality (Shapiro–Wilk test) or equal variance of nested data were not met, we used hierarchical blocked bootstrapping or permutation testing. For transcriptomic data, differential expression analysis was performed using the Wald test, with FDR controlled using the Benjamini–Hochberg correction. For comparing two cumulative distributions, Kolmogorov–Smirnov tests were used. For comparison of Gaussian fits, two-sided Wald *z*-tests, with Holm correction, were used.

Power calculation was performed to determine sample size. Given the technical complexity of some of the experiments, sample sizes are necessarily limited; nonetheless, the reproducibility of the effects across animals and modalities collectively support the conclusions.

### Reporting summary

Further information on research design is available in the Nature Portfolio Reporting Summary linked to this article.

## Online content

Any methods, additional references, Nature Portfolio reporting summaries, source data, extended data, supplementary information, acknowledgements, peer review information; details of author contributions and competing interests; and statements of data and code availability are available at 10.1038/s41586-026-10414-w.

## Supplementary information

Supplementary Table 1 A table summarizing the statistical tests used throughout this study.

Reporting Summary

Supplementary Video 1 Microglial engulfment (unlesioned): 3D volume reconstruction of an IBA1^+^ microglia (blue), containing CD68^+^ lysosomes (red) and engulfed PSD95 (green) in the IO from an unlesioned control animal.

Supplementary Video 2 Microglial engulfment (7 d.p.l.): 3D volume reconstruction of an IBA1^+^ microglia (blue), containing CD68^+^ lysosomes (red) and engulfed PSD95 (green) in the IO from a 7 d.p.l. animal.

Supplementary Video 3 Microglial engulfment (14 d.p.l.): 3D volume reconstruction of an IBA1^+^ microglia (blue), containing CD68^+^ lysosomes (red) and engulfed PSD95 (green) in the IO from a 14 d.p.l. animal.

## Source data

Source Data Figs. 1–5 and Source Data Extended Data Figs. 1–7, 9 and 10.

## Data Availability

All sequencing data have been deposited in NCBI’s Gene Expression Omnibus and are accessible through GEO series accession number GSE274050 (bulk RNA data) and series accession number GSE311781 (DBiT-seq). The *R. norvegicus* reference genome used to align bulk RNA-seq and DBiT-seq reads (mRatBN7.2, RefSeq annotation, NCBI: GCF_015227675.2) is available online. The scRNA-seq dataset used to deconvolute the bulk RNA-seq experiment is available online (https://cells.ucsc.edu/?ds=mouse-nervous-system). Source data are provided with this paper.

## Code availability

The codes used for RNA-seq deconvolution and DBit-seq analysis are available at GitHub (https://github.com/Castelo-Branco-lab/Karadottir_DBiT_2025).
