# Supplementary material for: Focal white matter lesions drive grey matter inflammation and synapse loss
Source: Nature. 2026 Apr 22;654(8120):1033–43. doi: 10.1038/s41586-026-10414-w (PMC13293868; doi:10.1038/s41586-026-10414-w)
Supplement: Supplementary file 2 — Reporting Summary [file 41586_2026_10414_MOESM2_ESM.pdf]

nature portfolio

Corresponding author(s): Ragnhildur T. Káradóttir

Last updated by author(s): Feb 12, 2026

Reporting Summary

Nature Portfolio wishes to improve the reproducibility of the work that we publish. This form provides structure for consistency and transparency in reporting. For further information on Nature Portfolio policies, see our Editorial Policies and the Editorial Policy Checklist.

Statistics

For all statistical analyses, confirm that the following items are present in the figure legend, table legend, main text, or Methods section.

n/a	Confirmed	
☐	☒	The exact sample size (*n*) for each experimental group/condition, given as a discrete number and unit of measurement
☐	☒	A statement on whether measurements were taken from distinct samples or whether the same sample was measured repeatedly
☐	☒	The statistical test(s) used AND whether they are one- or two-sided<br>*Only common tests should be described solely by name; describe more complex techniques in the Methods section.*
☐	☒	A description of all covariates tested
☐	☒	A description of any assumptions or corrections, such as tests of normality and adjustment for multiple comparisons
☐	☒	A full description of the statistical parameters including central tendency (e.g. means) or other basic estimates (e.g. regression coefficient) AND variation (e.g. standard deviation) or associated estimates of uncertainty (e.g. confidence intervals)
☐	☒	For null hypothesis testing, the test statistic (e.g. *F*, *t*, *r*) with confidence intervals, effect sizes, degrees of freedom and *P* value noted<br>*Give P values as exact values whenever suitable.*
☒	☐	For Bayesian analysis, information on the choice of priors and Markov chain Monte Carlo settings
☐	☒	For hierarchical and complex designs, identification of the appropriate level for tests and full reporting of outcomes
☐	☒	Estimates of effect sizes (e.g. Cohen's *d*, Pearson's *r*), indicating how they were calculated

Our web collection on statistics for biologists contains articles on many of the points above.

Software and code

Policy information about availability of computer code

Data collection	FACS FACSDIVA 9.0.1<br>Zeiss LSM 980 with a Airyscan 2, Zeiss Cell Observer, Zen blue 3.7.97.0.7000<br>Leica SP8 confocal microscope (LASX)<br>RNAseq- SMARTer Stranded Total RNA-Seq Pico input v3<br>Neurophotometrics FP3002 console
Data analysis	GraphPad Prism 10.2.3 and 10.5.0 for Windows, GraphPad Software, Boston, Massachusetts USA, www.graphpad.com<br>Imaris (Bitplane) versions 9.1 and 10.2<br>ImageJ (v1.54p)<br>Python (v3.11.13)<br>Quantification of synaptic puncta density was on ImageJ, using SynapseJ (Manrique et al., 2021)<br>HALO 3.6.4134 (Indica labs)<br>Aiforia Create, Aiforia Technologies Oy, Helsinki, Finland, https://www.aiforia.com/)<br>Bulk RNA-seq deconvolution used R (v4.5.0) , MuSiC R package (https://github.com/xuranw/MuSiC). Heat maps were plotted using ComplexHeatmap R package (https://github.com/jokergoo/ComplexHeatmap), Go analysis by EnrichR 3.4 package (https://www.rdocumentation.org/packages/enrichR/versions/3.4).<br>Deconvolution code is available here https://github.com/Castelo-Branco-lab/Karadottir_DBiT_2025<br>DBiTSeq data were processed and analysed using R (v4.3.3) with imager package (v1.0.2), Snakemake (v7.24.0) with bbduk (v39.01), cellranger count (v7.1.0), Seurat object (v5.1.0), FNN package (v1.1.4.1), igraph package (v2.1.4).<br>For fiber photometry data, normalized dF/F was calculated on Matlab (R2025a), using a published script (Martianova et al., 2019)<br>Mitochondria analysis images were deconvolved using Huygens Professional (https://svi.nl/Huygens-Professional), and analysed using the

Mitochondria Analyzer plugin on FIJI (v1.54p).

For manuscripts utilizing custom algorithms or software that are central to the research but not yet described in published literature, software must be made available to editors and reviewers. We strongly encourage code deposition in a community repository (e.g. GitHub). See the Nature Portfolio guidelines for submitting code & software for further information.

Data

Policy information about availability of data

All manuscripts must include a data availability statement. This statement should provide the following information, where applicable:

- Accession codes, unique identifiers, or web links for publicly available datasets
- A description of any restrictions on data availability
- For clinical datasets or third party data, please ensure that the statement adheres to our policy

All sequencing data have been deposited in NCBI's Gene Expression Omnibus and are accessible through GEO series accession number GSE274050 (https://www.ncbi.nlm.nih. gov/geo/query/acc.cgi?acc=GSE274050) (Bulk RNAdata) and series accession number GSE311781 (https://www.ncbi.nlm.nih.gov/geo/query/acc.cgi?acc= GSE311781) (DBiT-seq). The Rattus norvegicus reference genome used to align Bulk RNAseq and DBiT-seq reads (mRatBN7.2, RefSeq annotation, NCBI, accession number GCF_015227675.2) is available here: https://www.ncbi.nlm.nih.gov/datasets/genome/GCF_015227675.2. The single-cell RNAseq dataset used to deconvolute the bulk RNAseq is available here: https://cells.ucsc.edu/?ds=mouse-nervous-system.

Research involving human participants, their data, or biological material

Policy information about studies with human participants or human data. See also policy information about sex, gender (identity/presentation), and sexual orientation and race, ethnicity and racism.

Reporting on sex and gender	N/A
Reporting on race, ethnicity, or other socially relevant groupings	N/A
Population characteristics	N/A
Recruitment	N/A
Ethics oversight	N/A

Note that full information on the approval of the study protocol must also be provided in the manuscript.

Field-specific reporting

Please select the one below that is the best fit for your research. If you are not sure, read the appropriate sections before making your selection.

☒ Life sciences ☐ Behavioural & social sciences ☐ Ecological, evolutionary & environmental sciences

For a reference copy of the document with all sections, see nature.com/documents/nr-reporting-summary-flat.pdf

Life sciences study design

All studies must disclose on these points even when the disclosure is negative.

Sample size	For remyelination, histological studies and in vivo calcium and ATP recordings, sample size was based on previous datasets (Gautier et al., 2015; Salinas et al., 2023) which overall have a Cohen's effect size d of 4. Thus, a sample size of n=3 for control and n=3 for experimental groups provides a statistical power greater than 0.8 at an alpha level of 0.05. For morphological analysis, engulfment quantification and mitochondrial studies, a dataset of n=3 or more cells analysed from 3 biological replicates per group is required to achieve a statistical power greater than 0.8 at an alpha level of 0.05. For bulk RNAseq and DBiT studies, sample size was determined based on the literature (Spitzer et al., 2019; Zhang et al., 2023).
Data exclusions	28dpl DBiT-seq samples did not reach minimum tissue quality (loss of structural/spatial information) and were excluded. Areas containing the surgical needle track were excluded from the analysis, as the inflammatory reaction around the needle track is a confounder factor.
Replication	All data was replicated and individual replicates are represented within the figures. In the figure legends the number of biological replicates is explained. The number of replicates is also indicated in the supplementary table.
Randomization	All animals were randomized into experimental groups. For immunohistochemical analysis, samples were allocated by randomly selecting brain slices containing the regions of interest from each experimental group.
Blinding	Experimenters were not blinded to experimental conditions during animal surgery and recovery, as lesioned animals, but not unlesioned controls, can develop side effects that require enhanced monitoring. However, subsequent tissue processing, imaging and quantification

were performed blindly and validated by more than two experimenters. Experiments that did not require animal surgery were performed blindly at all stages of data collection and quantification.

Reporting for specific materials, systems and methods

We require information from authors about some types of materials, experimental systems and methods used in many studies. Here, indicate whether each material, system or method listed is relevant to your study. If you are not sure if a list item applies to your research, read the appropriate section before selecting a response.

Materials & experimental systems

n/a	Involved in the study
☐	☒ Antibodies
☒	☐ Eukaryotic cell lines
☒	☐ Palaeontology and archaeology
☐	☒ Animals and other organisms
☒	☐ Clinical data
☒	☐ Dual use research of concern
☒	☐ Plants

Methods

n/a	Involved in the study
☒	☐ ChIP-seq
☐	☒ Flow cytometry
☒	☐ MRI-based neuroimaging

Antibodies

Antibodies used

For Immunofluorescence:
Primary Antibodies:
Abcam: rabbit CD68 (ab125212; 1:100); mouse calbindin (CB-955, ab82812; 1:100); rabbit calbindin (EP3478, ab108404; 1:200); rabbit C1q (4.8, ab182451; 1:100); mouse NG2 (132.38, ab50009; 1:200); rabbit COX-IV (ab16056; 1:100); chicken anti m-cherry (ab205402; 1:500); chicken anti-GFP (ab13970; 1:1000); rabbit GABABr1 (EPR22954-47, ab238130; 1:100); mouse P62 (2C11, ab56416; 1:100); Chicken MAP2 (ab5392; 1:2000); mouse bassoon (SAP7F407, ab82958; 1:100)
Antibodies.com: chicken GFAP (A85307; 1:300); Goat IBA1 (a82670; 1:100)
Wako: rabbit IBA1 (Cat#019-19741; 1:500)
Merck Millipore: mouse PSD95 (6G6-1C9, MAB1596; 1:100); guinea pig vGLUT2 (AB2251-I; 1:500), Rabbit Olig2 (AB9610; 1:300).
Synaptic Systems: mouse bassoon (SAP7F407; 1:100)
R&D Systems: goat LDLR (AF2255; 1:100)

Secondary antibodies (All secondary antibodies were used at 1:500 dilution.):
ThermoFisher: Goat anti rabbit Alexa Fluor 488 (A32731); Goat anti rabbit Alexa Fluor 568 (A11036); Goat anti rabbit Alexa Fluor 647 (A21247); Goat anti mouse Alexa Fluor 750 (A21037); Goat anti mouse Alexa Fluor 647 (A21242); Goat anti mouse Alexa Fluor 555 (A21424); Goat anti mouse Alexa Fluor 488 (A11029); Goat anti mouse Alexa Fluor 568 (A11031); Donkey anti goat Alexa Fluor 633 (A21082; Donkey anti Rabbit Alexa Fluor 488 (A-21206)
Abcam: Goat anti hamster Alexa Fluor 488 (ab173003), Goat anti guinea pig Alexa Fluor 647 (ab150187), Goat anti chicken Alexa Fluor 568 (ab175477)

Validation

All antibodies listed above are commercially available and have been validated by the manufacturers and previous publications. For further details please see below:

rabbit CD68 (Abcam, ab125212; 1:100): validated for Western Blot, IHC-P. Suitable for Mouse, Rat - https://www.abcam.com/en-us/products/primary-antibodies/cd68-antibody-ab125212

mouse calbindin (Abcam, ab82812; 1:100): validated for Western Blot, IHC-P. Suitable for Cow, Human, Mouse, Rat - https://www.abcam.com/en-us/products/primary-antibodies/calbindin-antibody-cb-955-ab82812

rabbit calbindin (Abcam, ab108404; 1:200): validated for Western Blot, Flow Cytometry (Intra), Flow Cytometry, IHC-P, IHC-Fr, ICC/IF, mIHC. Suitable for Human, Mouse, Rat - https://www.abcam.com/en-us/products/primary-antibodies/calbindin-antibody-ep3478-ab108404

rabbit C1q (Abcam, ab182451; 1:100): validated for IHC-Fr. Suitable for Mouse, Rat - https://www.abcam.com/en-us/products/primary-antibodies/c1q-antibody-48-ab182451

mouse Ng2 (Abcam, ab50009; 1:200) – validated for WB and reacts with Rat samples. - https://www.abcam.com/en-us/products/primary-antibodies/ng2-antibody-13238-ab50009

rabbit COX-IV (Abcam, ab16056; 1:100): validated for Western Blot, IHC-P, IHC-Fr, ICC/IF. Suitable for Human, Mouse, Rat - https://www.abcam.com/en-us/products/primary-antibodies/cox-iv-antibody-mitochondrial-loading-control-ab16056

chicken anti m-cherry (Abcam, ab205402; 1:500): validated for Western Blot, ICC/IF. Suitable for Mouse, Rat - https://www.abcam.com/en-us/products/primary-antibodies/mcherry-antibody-ab205402

chicken anti-GFP (Abcam, ab13970; 1:1000): validated for Western Blot, ICC/IF. Suitable for Mouse, Rat - https://www.abcam.com/en-us/products/primary-antibodies/gfp-antibody-ab13970

rabbit GabaArg1 (Abcam, ab238130; 1:100): Suitable for WB, IHC-Fr, IHC-P and reacts with Mouse, Rat, Human samples - https://www.abcam.com/en-us/products/primary-antibodies/gaba-b-receptor-1-antibody-epr22954-47-ab238130

mouse P62 (Abcam, ab56416; 1:100): validated for Western Blot, Flow Cytometry, IP, IHC-P, ICC/IF. Suitable for Human, Rat - https://www.abcam.com/en-us/products/primary-antibodies/sqstm1-p62-antibody-2c11-bsa-and-azide-free-ab56416

chicken GFAP (Antibodies.com, A85307; 1:300): validated for WB, ICC/IF and IHC. Suitable for Human, Horse, Cow, Porcine, Rat, Mouse - https://www.antibodies.com/ catalog/primary-antibodies/gfap-antibody-a85307

goat IBA1 (Antibodies.com, a82670; 1:100): validated for ELISA, WB and IHC. Suitable for Human, Mouse, Rat - https://www.antibodies.com/catalog/primary-antibodies/iba1-antibody-a82670.

chicken MAP2 (Abcam, ab5392; 1:2000): validated for Western Blot, IHC-P, ICC/IF. Suitable for Mouse, Rat - https://www.abcam.com/en-us/products/primary-antibodies/map2-antibody-neuronal-marker-ab5392

rabbit Olig2 (Merck Millipore, AB9610; 1:300): validated for Western Blot, ICC, IHC (p), IP. Suitable for Mouse, Human, Rat - https://www.sigmaaldrich.com/GB/en/product/mm/ab9610

rabbit IBA1 (Wako, 019-19741; 1:500): validated for ICC, IHC. Suitable for Mouse, Rat - https://labchem-wako.fujifilm.com/us/product/detail/W01W0101-1974.html
mouse PSD95 (Merck Millipore, MAB1596; 1:100): validated for WB, ICC, IHC. Suitable for Mouse, Rat.https://www.sigmaaldrich.com/GB/en/product/mm/mab1596

guinea pig vGLUT2 (Merck Millipore, AB2251-I; 1:500): validated for WB, IHC. Suitable for Mouse, Rat - https://www.sigmaaldrich.com/GB/en/product/mm/ab2251i

mouse bassoon (Synaptic Systems, SAP7F407; 1:100): validated for Western Blot, IHC-P, ICC/IF. Suitable for Human, Mouse, Rat - https://www.abcam.com/en-us/products/primary-antibodies/bassoon-bsn-antibody-sap7f407-ab82958

goat LDLR (R&D Systems, AF2255; 1:100): validated for Immunohistochemistry, Western Blot, Blockade of Receptor-ligand Interaction, Flow Cytometry, CyTOF-ready. Suitable for Mouse, Rat. https://www.rndsystems.com/products/mouse-ldlr-antibody_af2255

Animals and other research organisms

Policy information about studies involving animals; ARRIVE guidelines recommended for reporting animal research, and Sex and Gender in Research

Laboratory animals	Surgeries used Sprague Dawley rats purchased from Charles River, 9-12 weeks or 18 months old; or 18 month old Sox10:dsRed rats, 10.1016/j.stemcr.2017.09.005; and 3-4 month old mice, bred in house (Tg(Pdgfra-cre/ERT2)1Wdr or Tg(Pdgfra-cre/ERT2)1Wdr:Mapttm2Arbr); or 3-4 month-old c57bl/6 mice purchased from Charles River. Tissue culture used Sprague Dawley rats, P0-2. All animals were maintained under a 12 h light:12 h dark cycle with food and water supplied ad libitum.
Wild animals	The study did not involve wild animals.
Reporting on sex	Animal surgeries were performed on females exclusively. Tissue cultures were from pooled mixed sex litters.
Field-collected samples	No samples were collected in the field.
Ethics oversight	Experiments were performed in accordance with the EU guidelines for the care and use of laboratory animals, and with the guidelines of the UK Animals (Scientific Procedures) Act 1986 and subsequent amendments. Use of animals in this project was approved by the Animal Welfare and Ethical Review Body for the University of Cambridge and carried out under the terms of UK Home Office Licenses PP4353554, P9B1FBC4B and 70/7715, and the Animal Welfare and Ethical Review Body by the Icelandic Food and Veterinary Authority.

Note that full information on the approval of the study protocol must also be provided in the manuscript.

Plants

Seed stocks

Report on the source of all seed stocks or other plant material used. If applicable, state the seed stock centre and catalogue number. If plant specimens were collected from the field, describe the collection location, date and sampling procedures.

Novel plant genotypes

Describe the methods by which all novel plant genotypes were produced. This includes those generated by transgenic approaches, gene editing, chemical/radiation-based mutagenesis and hybridization. For transgenic lines, describe the transformation method, the number of independent lines analyzed and the generation upon which experiments were performed. For gene-edited lines, describe the editor used, the endogenous sequence targeted for editing, the targeting guide RNA sequence (if applicable) and how the editor was applied.

Authentication

Describe any authentication procedures for each seed stock used or novel genotype generated. Describe any experiments used to assess the effect of a mutation and, where applicable, how potential secondary effects (e.g. second site T-DNA insertions, mosiacism, off-target gene editing) were examined.

Flow Cytometry

Plots

Confirm that:

☒ The axis labels state the marker and fluorochrome used (e.g. CD4-FITC).

☒ The axis scales are clearly visible. Include numbers along axes only for bottom left plot of group (a 'group' is an analysis of identical markers).

☒ All plots are contour plots with outliers or pseudocolor plots.

☒ A numerical value for number of cells or percentage (with statistics) is provided.

Methodology

Sample preparation

Micro dissected inferior olive tissue was digested with papain solution in DMEM buffer for 30-45 minutes at 37°C. Cells were triturated to single cells after blocking papain activity with ice cold soybean trypsin inhibitor solution. After filtering remaining cell aggregates with 40um strainer, cells were labelled in PBS without Ca and Mg supplemented with 0.5% BSA fraction V and separated from myelin debris with myelin removal microbead kit from Miltenyi. Dead and dying cells were labelled with propidium iodide for 5 minutes in PBS without Ca and Mg supplemented with 0.5% BSA fraction V before analysis. Cells from control cortical tissue were heat shocked at 45°C for 2 minutes for dead cell positive control before propidium iodide labelling.

Instrument

BD FACSAria Fusion Cell Sorter #P656700G50009

Software

FACSDIVA 9.0.1

Cell population abundance

Fast blue+ PI- cells ranged between 0.6 and 6%.

Gating strategy

Cells gate was defined by FSC-A and SSC-A values. Singlets gate was defined by FSC-W over FSC-A value size. Live cell gate was defined by propidium iodide signal based on untreated or heat shocked cortical cells from the same rat brain as the inferior olive cells. Fast Blue+ cell gate was defined in PI- live cortical cells.

☒ Tick this box to confirm that a figure exemplifying the gating strategy is provided in the Supplementary Information.
